# Colibactin Contributes to the Hypervirulence of *pks*^+^ K1 CC23 *Klebsiella pneumoniae* in Mouse Meningitis Infections

**DOI:** 10.3389/fcimb.2017.00103

**Published:** 2017-03-31

**Authors:** Min-Chi Lu, Ying-Tsong Chen, Ming-Ko Chiang, Yao-Chen Wang, Pei-Yi Hsiao, Yi-Jhen Huang, Ching-Ting Lin, Ching-Chang Cheng, Chih-Lung Liang, Yi-Chyi Lai

**Affiliations:** ^1^Division of Infectious Diseases, Department of Internal Medicine, China Medical University HospitalTaichung, Taiwan; ^2^Department of Microbiology and Immunology, School of Medicine, China Medical UniversityTaichung, Taiwan; ^3^Institute of Molecular and Genomic Medicine, National Health Research InstitutesMiaoli County, Taiwan; ^4^Institute of Genomics and Bioinformatics, National Chung Hsing UniversityTaichung, Taiwan; ^5^Department of Life Science, National Chung Cheng UniversityChia-Yi County, Taiwan; ^6^Department of Internal Medicine, Chung Shan Medical University HospitalTaichung, Taiwan; ^7^Department of Microbiology and Immunology, Chung Shan Medical UniversityTaichung, Taiwan; ^8^Graduate Institute of Chinese Medicine, School of Chinese Medicine, China Medical UniversityTaichung, Taiwan; ^9^Laboratory Animal Service Center, China Medical UniversityTaichung, Taiwan

**Keywords:** *Klebsiella pneumoniae*, K1, CC23, colibactin, meningitis

## Abstract

*Klebsiella pneumoniae* is the most common pathogen of community-acquired meningitis in Taiwan. However, the lack of a physiologically relevant meningitis model for *K. pneumoniae* has impeded research into its pathogenesis mechanism. Based on the core genome MLST analyses, the hypervirulent K1 *K. pneumoniae* strains, which are etiologically implicated in adult meningitis, mostly belong to a single clonal complex, CC23. Some K1 CC23 *K. pneumoniae* strains carry a gene cluster responsible for colibactin production. Colibactin is a small genotoxic molecule biosynthesized by an NRPS-PKS complex, which is encoded by genes located on the *pks* island. Compared to other hypervirulent *K. pneumoniae* which primarily infect the liver, the colibactin-producing (*pks*^+^) K1 CC23 strains had significant tropism toward the brain of BALB/c mice. We aimed in this study to develop a physiologically relevant meningitis model with the use of *pks*^+^ K1 CC23 *K. pneumoniae*. Acute meningitis was successfully induced in adult BALB/c male mice through orogastric, intranasal, and intravenous inoculation of *pks*^+^ K1 CC23 *K. pneumoniae*. Besides the typical symptoms of bacterial meningitis, severe DNA damages, and caspase 3-independent cell death were elicited by the colibactin-producing K1 CC23 *K. pneumoniae* strain. The deletion of *clbA*, which abolished the production of colibactin, substantially hindered *K. pneumoniae* hypervirulence in the key pathogenic steps toward the development of meningitis. Our findings collectively demonstrated that colibactin was necessary but not sufficient for the meningeal tropism of *pks*^+^ K1 CC23 *K. pneumoniae*, and the mouse model established in this study can be applied to identify other virulence factors participating in the development of this life-threatening disease.

## Introduction

*Klebsiella pneumoniae* has become a global threat to human health during the last decade. Because of the emergence of multidrug resistance strains, infections caused by this bacterium are problematic in hospitals. Based on the virulence, *K. pneumoniae* strains can be divided into two groups, “classic” or “hypervirulent” (Shon et al., 2013). Most of the hospital-acquired infections are caused by “classic” *K. pneumoniae* strains, which exhibit restricted virulence and frequently acquire resistance to antibiotics, such as ESBL (extended-spectrum β-lactamases) or carbapenem-resistance. From the mid-1980s, the emergence of “hypervirulent” *K. pneumoniae* strains has been noted, particularly in Taiwan. With invasive virulence, these strains cause severe community-acquired infections, such as pyogenic liver abscess, meningitis, and endophthalmitis (Wang et al., 1998; Fung et al., 2002). Of the 78 capsular serotypes, hypervirulent *K. pneumoniae* strains are frequently serotyped K1 or K2 (Nassif and Sansonetti, 1986; Yeh et al., 2007). However, some of the “classic” *K. pneumoniae* strains are also K1 or K2. Recent phylogenetic analyses using a 694-gene core genome MLST (multilocus sequence typing) scheme revealed that the hypervirulent K1 *K. pneumoniae* strains mostly belonged to a single clonal complex, CC23 (Brisse et al., 2009; Struve et al., 2015). This indicates that the clonal lineage CC23 has a specific genetic background conferring hypervirulence and fitness. Variants of a genomic island carrying genes responsible for biosynthesis of yersiniabactin, colibactin, and microcin E492 were uniquely identified in the genomes of K1 CC23 hypervirulent *K. pneumoniae* strains (Bialek-Davenet et al., 2014; Struve et al., 2015).

Bacterial meningitis is one of the most devastating brain diseases. Hypervirulent *K. pneumoniae*, particularly K1 CC23 type, is the most commonly implicated pathogen of adult bacterial meningitis (Fang et al., 2007; Chang et al., 2012). Usually, *K. pneumoniae* meningitis is a severe syndrome comprised of multiple septic metastatic lesions, including liver, eyes, lung, and kidney (Chang et al., 2012) and may occur in the early stage of hospital admission. The reported mortality rates of *K. pneumoniae* meningitis range from 33.3 to 48.5% (Tang et al., 1997; Fang et al., 2000, 2007; Lu et al., 2000). More than half of patients with *K. pneumoniae*-induced meningitis and brain abscess developed persistent neurological deficits after therapy (Fang et al., 2007). Despite the high mortality rates, research on the pathogenesis mechanism regarding *K. pneumoniae* meningitis is so far restricted.

*K. pneumoniae* 1084, a hypervirulent K1 isolate, carries a 208-kb genomic island and is phylogenically located in the clonal complex CC23. This 208-kb genomic island, named KPHPI208, is composed of eight genomic modules (Lai et al., 2014). The first genomic module contains genes ~100% identical to those of the *pks* island reported in newborn meningitis *Escherichia coli* strain IHE3034 (Nougayrede et al., 2006). The 54-kb *pks* island encodes a non-ribosomal peptide synthetase-polyketide synthase (NRPS-PKS) assembly line, responsible for the biosynthesis of colibactin, a small genotoxic metabolite. Transient infection with colibactin-producing *E. coli* strains induced host cellular DNA damages *in vitro* and *in vivo* (Nougayrede et al., 2006). Recent studies in *E. coli* have linked the colibactin production to long-term bacterial persistence in the gastrointestinal tract, colorectal tumorigenesis (Dalmasso et al., 2014; Raisch et al., 2014), and the development of neonatal systemic infection (McCarthy et al., 2015).

With the carriage of KPHPI208, *K. pneumoniae* 1084 is recognized as a *pks*^+^ K1 CC23 strain. In our previous study (Lai et al., 2014), we demonstrated that the deletion of *clbA*, which encoded a 4′-phosphopantetheinyl transferase (PPTase) essential for the synthesis of colibactin, significantly attenuated the capacity of *K. pneumoniae* 1084 to induce DNA damages *in vitro* and *in vivo*. In addition to the genotoxicity, we observed that the majority of BALB/c mice which were orogastrically inoculated with *K. pneumoniae* 1084 developed vital signs of meningitis during the course of the experiment, suggesting a meningeal tropism of this strain. Although the majority of hypervirulent *K. pneumoniae* strains can develop invasive infections from intestinal colonization, liver is the primary organ they attack. The tropism of *K. pneumoniae* 1084 toward the central nervous system suggested the presence of *pks*^+^ K1 CC23-specific factors contributing to bacterial meningitis. To get insights into the molecular mechanism, we first established physiologically relevant meningitis models with the use of *K. pneumoniae* 1084. Through orogastric and intranasal inoculation, *K. pneumoniae* 1084 induced meningitis at 5–7 days post-inoculation, suggesting an ability of this *pks*^+^ K1 CC23 strain to penetrate mucosal barriers. Resistance to the blood clearance and translocation across the blood-brain barrier allowed this strain to develop meningitis as soon as 24 h post-intravenous-inoculation. Typical symptoms of acute bacterial meningitis were revealed through the examination of the brain sections obtained from the *K. pneumoniae* 1084-infected mice. Besides, DNA damages and extensive cell death in the brain were also induced by this colibactin-producing strain. The full virulence for *K. pneumoniae* 1084 to cause meningitis required the involvement of colibactin. The *clbA* deletion mutant, ΔClbA, was significantly attenuated in the key pathogenic steps toward the development of meningitis.

## Materials and methods

### *K. pneumoniae* strains and growth conditions

*K. pneumoniae* 1084 was isolated from a diabetic patient with the bacteremic infections at a referral medical center in central Taiwan during 2002–2004 (Lin et al., 2011). This strain was K1 and phylogenically located in the clonal complex CC23 (Bialek-Davenet et al., 2014; Struve et al., 2015). *K. pneumoniae* 1084S was a streptomycin-resistant variant of 1084, exhibiting hypervirulence to male BALB/c mice. The genome of this strain was fully sequenced and deposited in GenBank (NC_018522.1). KPHPI208 is a 208-kb genomic island identified in *K. pneumoniae* 1084 (Lai et al., 2014). The first module of the eight genomic modules predicted in KPHPI208 contained genes ~100% identical to the *pks* colibactin gene cluster reported in *E. coli* IHE3034 (Nougayrede et al., 2006). The *clbA* deletion mutant of *K. pneumoniae* 1084S, named ΔClbA, and ΔClbA_pC (ΔClbA in-*trans* complemented with pYC502, the *clbA*-containing plasmid) were generated in our previous study (Lai et al., 2014). To avoid the problem derived from the loss of pYC502, we generated an in-*cis clbA* complement strain (named ΔClbA_cC), in which the full-length *clbA* gene was integrated into the *lacZ* locus of ΔClbA. Briefly, 1,200 bp DNA fragments flanking the insertion site within *lacZ* were amplified with specific primer sets, p548 (AGCCACACTCCTGACTTTCA)/p549 (AGCGGTTGCAGAGTTCATACCA) and p550 (TCGGCAACTTCGCCGATTACT)/p551 (AGTCCAGCGCCTGGGTATCA), and cloned into the suicide vector, pKAS46 (Skorupski and Taylor, 1996). The resulting construct was named pYC520. A full-length *clbA* gene (793 bp) was amplified with p552 (GGTACCACAAGCTCGGAATACGAATCA)/p553 (GAGCTCAATTCTGCCCATTTGACGA), digested with *Kpn*I/*Sac*I, and cloned into the inserts on pYC520. The resulting construct pYC521 in *E. coli* S17-1 λ *pir* was subsequently mobilized to ΔClbA via conjugation. Kanamycin-resistant transconjugants were selected, propagated in LB, and then subjected to streptomycin selection (500 μg/ml). After the occurrence of double cross-over, colonies showing resistance to streptomycin and susceptibility to kanamycin were isolated. The insertion of *clbA* gene into the *lacZ* locus was verified by PCR. *K. pneumoniae* 1084 and its variants were maintained in Luria-Bertani (LB) broth. Mid-log cultures with a determined density of CFU/ml were used for all the infection experiments.

### Ethics statement

All the animal experiments were performed under the recommendation in the Guide for the Care and Use of Laboratory Animals of the National Laboratory Animal Center (NLAC, Taiwan), and all surgery and treatment were performed under anesthesia with all efforts to minimize the suffering of animals. The animal protocols were approved by Chung Shan Medical University Experimental Animal Center (Permit number: 1392).

### Mouse infections

Male BALB/c mice were purchased from National Laboratory Animal Center (NLAC, Taiwan) at the age of 7-wk-old and allowed to acclimatize in the animal house for 1 week before experiments. Meningitis was induced through three different administration routes. In the first model, groups of 8-wk old BALB/c male mice received streptomycin (500 μg/ml) in drinking water for 3 days prior to infection. Two milliliters of mid-log phase culture of a particular strain were pellet by centrifugation at 13,000 rpm for 5 min and then resuspended in 200 μL of saline. Two hundred microliters of saline containing 1 × 10^9^ CFU of *K. pneumoniae* 1084S, ΔClbA, or ΔClbA_pC were orogastrically inoculated into individual groups of mice through a 20-gauge × 1.5-inch feeding needle. Feces were daily collected from each of the mice and dissolved in saline for CFU determination. At the 7th day post-inoculation, the infected mice were sacrificed. Mouse tissues, including intestines, liver, spleen, brain, and blood were retrieved for bacterial enumeration, histopathology examination, and immunohistochemistry analysis. In the second model, groups of 8-wk old BALB/c male mice were intranasally inoculated through a microliter pipette tip with 30 μL of saline containing 1 × 10^6^ CFU of *K. pneumoniae* 1084S, ΔClbA, or ΔClbA_cC. The survival rate of the inoculated mice was daily monitored and determined by Kaplan-Meier analysis using Prism5 for Windows (GraphPad). At the 5th day post-inoculation, the infected mice which survived the experimental period were sacrificed. Brain, lungs, spleen, and blood were harvested for enumeration of CFU. To bypass the intestinal or nasopharyngeal barrier, *K. pneumoniae* 1084S, ΔClbA, and ΔClbA_cC were, respectively, injected by the intravenous route at an inoculum of 1 × 10^6^ CFU. At 24 h post-inoculation, brains were harvested for CFU determination, histopathology examination, immunohistochemistry analysis, and the measurement of inflammatory cytokines.

### Brain histopathology and immunochemistry analysis

At indicative time points, the PBS-inoculated control and the *K. pneumoniae*-infected mice were sacrificed under anesthesia. Brains were carefully harvested, immediately fixed in 10% of formalin, and processed for paraffin embedding. Hematoxylin and eosin (H/E) stained brain sections were examined by experienced pathologists for evaluation of meningitis and brain damages. For the detection of MMP-9, lipocalin-2 (LCN2), cleaved caspase 3, and phospho-H2AX, the paraffin-embedded brain sections were de-waxed with xylene, rehydrated in graded alcohol, underwent antigen-retrieval with 0.01 M of citric buffer (pH 6.0), blocked, and hybridized with specific antibodies against MMP9 (Abcam), LCN2 (EMD Millipore), cleaved caspase 3 (Cell Signaling Technique; CST), and phospho-H2AX (CST), respectively. All the immunohistochemistry analyses were performed with the use of NovoLinkTM Polymer Detection System (Leica Biosystem) according to the manufacturer's protocol. The cell death of brain tissues was examined by TUNEL (Terminal deoxynucleotidyl transferase-mediated dUTP nick-end labeling) assay with the use of *In situ* Cell Death Detection kit (Roche). Whole tissue sections were examined by Zeiss Axio microscope and scanned using the TissueFAXS plus system (TissueGnostics GmbH). The acquired images were analyzed and positive signals for all the immunohistochemical sections were respectively quantified using the HistoQuest Software provided by TissueGnostics GmbH (Vienna, Austria).

### Bio-plex mouse cytokine assay

The brain retrieved from all experimental groups was homogenized with lysis buffer (1% Triton X-100 and complete protease and phosphatase inhibitors in PBS). The protein concentration of resulting brain lysate was quantified with BCA Protein Assay Kit (Thermo Scientific Pierce). Quantitation of interleukin (IL)-1α, IL-1β, IL-2, IL-3, IL-4, IL-5, IL-6, IL-9, IL-10, IL-12 (p40), IL-12 (p70), IL-13, IL-17, eotaxin, G-CSF, GM-CSF, IFN-g, KC, MCP-1, MIP-1α, MIP-1β, RANTES, and TNF-α in the brain lysates (100 μg of total proteins) was performed by the use of Bio-Plex Multiplex Immunoassays (Bio-Rad) according to the instruction manual.

### Statistical analysis

The difference between the indicative groups was determined by Student's *t*-test or Mann-Whitney test. Statistical significance was based on a one-tailed *p* < 0.05.

## Results

### The requirement of colibactin for the full virulence of *K. pneumoniae* 1084, a *pks*^+^ K1 CC23 strain, to establish systemic infections from the intestinal tract

**Figure 1:**
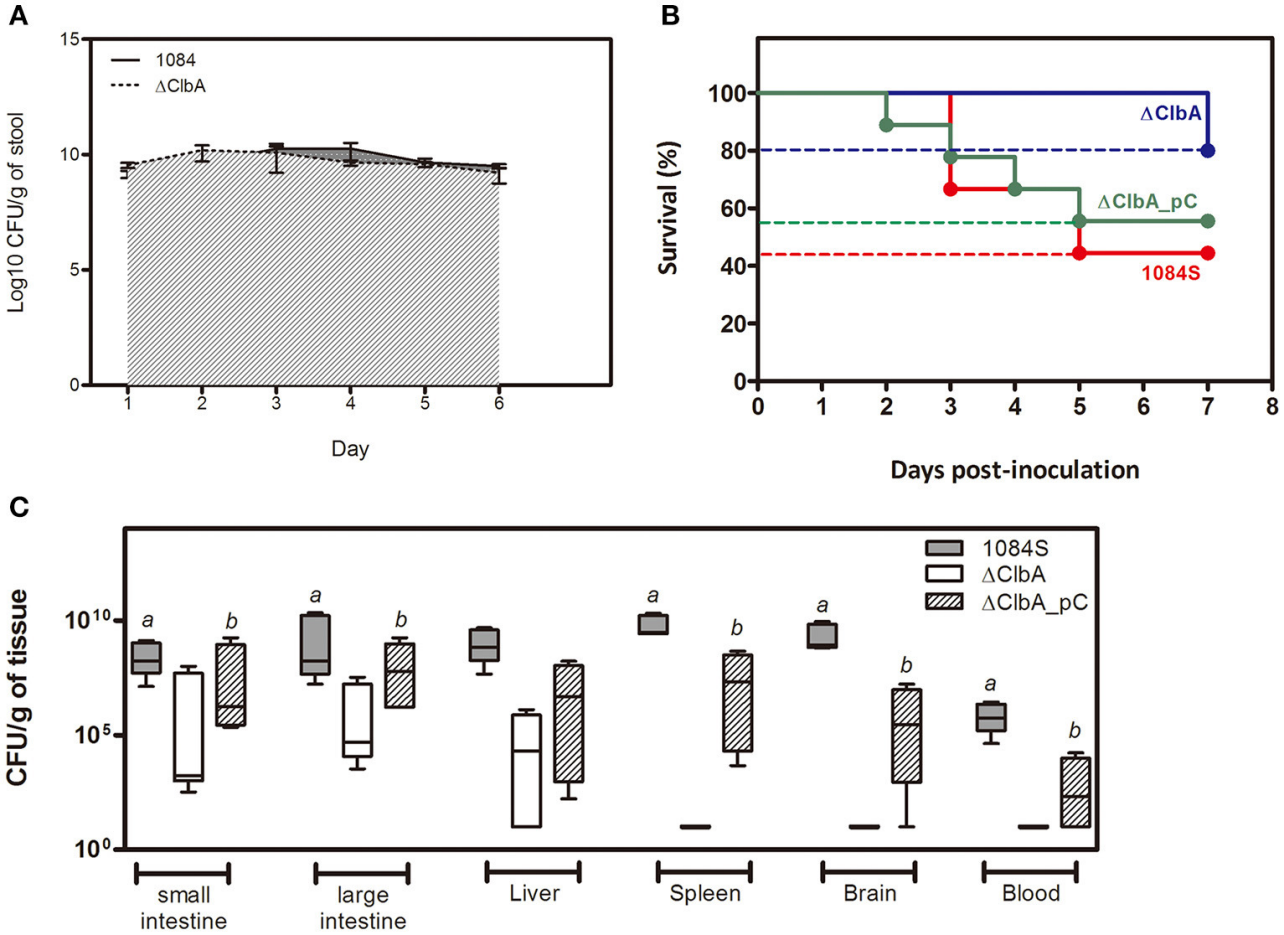
**Involvement of colibactin in intestinal colonization and the capacity of ***K. pneumoniae*** 1084, a ***pks***^**+**^ K1 CC23 hypervirulent strain, to establish systemic infections**. Groups of 8-wk old BALB/c male mice were orogastrically inoculated with 1 × 10^9^ CFU of *K. pneumoniae* 1084S, ΔClbA, or ΔClbA_pC. **(A)** Fecal shedding of 1084S (solid line) and ΔClbA (slash line) presented as a geometric mean of CFU per gram of stool, determined daily by a plate count method. **(B)** Survival rates of 1084S- (*n* = 9; red line), ΔClbA- (*n* = 6; blue line), or ΔClbA_pC-inoculated (*n* = 9; green line) mice determined by Kaplan-Meier analysis using Prism5 (GraphPad) are shown. **(C)** At the 7th day post-inoculation, the infected mice which survived the experimental period were sacrificed. The mucosa of the small and large intestines, liver, spleen, brain, and blood were retrieved and homogenized for the determination of bacterial loads for the 1084S- (dark gray), ΔClbA- (white), and ΔClbA_pC- (slash) group. CFU per gram of tissues is presented in a box and whisker plot. The edges of each box are the 25th and 75th percentiles, and the middle is the median. Lower case letter “*a*” and “*b*” represents statistical significance of CFUg^−1^, *p* < 0.05 (one-tailed) determined by Mann-Whitney test, between 1084S/ΔClbA and ΔClbA/ΔClbA_pC, respectively.

An intestinal portal of entry has been suggested for the development of invasive infections, such as liver abscess and meningitis, by hypervirulent *K. pneumoniae* strains (Tu et al., 2009; Fung et al., 2012; Lin et al., 2013; Hsu et al., 2015). To examine whether colibactin contributed to the development of invasive infections caused by *K. pneumoniae* 1084 via the intestinal route, we used 1 × 10^9^ CFU of *K. pneumoniae* 1084S, ΔClbA (the *clbA* deletion mutant of 1084S), and ΔClbA_pC (ΔClbA complemented with pYC502, the *clbA*-containing plasmid) to challenge groups of BALB/c mice via an orogastric inoculation route. During the first 6 days postinoculation, the bacterial counts of ΔClbA in the intestinal lumen were maintained similarly as that of *K. pneumoniae* 1084S. The quantitative CFU measurements of the shedding feces from both groups exceeded 10^9^ CFUg^−1^ (Figure 1A). However, the number of ΔClbA colonized the mucosa of small and large intestines was significantly reduced >1,000-fold when compared to that of *K. pneumoniae* 1084S (Figure 1C). This result suggested that colibactin was required for *K. pneumoniae* to establish a reservoir in the intestinal mucosa. Although *K. pneumoniae* resided in both the lumen and mucosa of intestines, only those colonizing the mucosa had a chance to invade the intestinal barrier to the developing of systemic infections. Therefore, the negative detection of ΔClbA in most of the extraintestinal organs (Figure 1C) was probably due to the reduced capacity of ΔClbA in the persistence of colonization of intestinal mucosa. In-*trans*-complementation of an entire *clbA* gene restored the ability of intestinal colonization and systemic dissemination (Figures 1B,C). These results suggested that the production of colibactin was required for *K. pneumoniae* 1084 to establish intestinal colonization and subsequent systemic infections.

### Induction of meningitis following *K. pneumoniae* 1084 dissemination through intestinal/respiratory route

**Figure 2:**
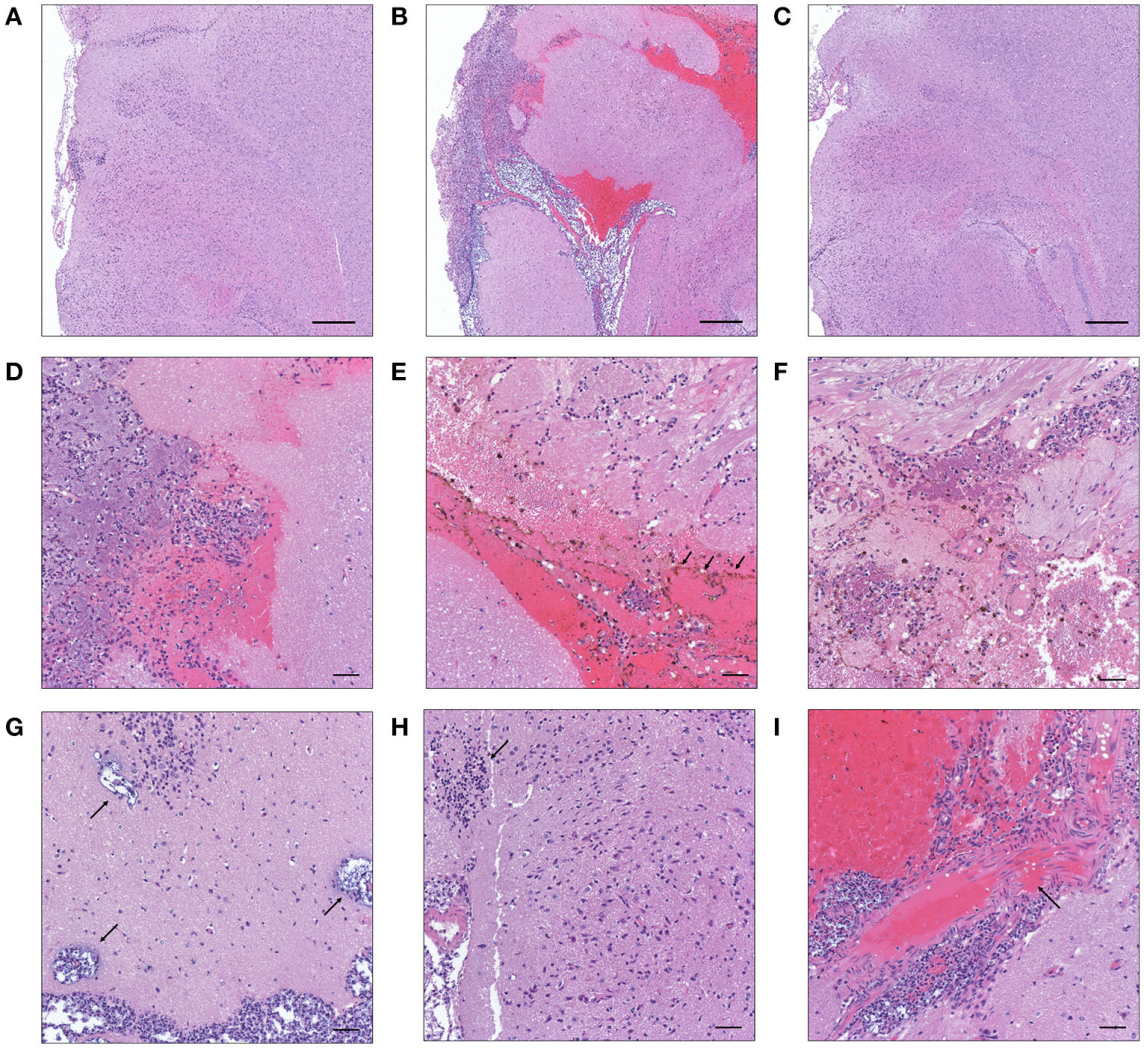
**Histopathological characteristics of ***K. pneumoniae*** 1084S-induced meningitis in adult BALB/c male mice**. Representative images of brain sections retrieved from the control mice **(A)** and the mice at 7 days post-orogastric-inoculation with 1 × 10^9^ CFU of *K. pneumoniae* 1084S **(B)** or with ΔClbA **(C)** are shown. In subarachnoid space of the 1084S-infected mice, numerous *K. pneumoniae* accumulated and neutrophils were recruited **(D)**. Upon infection, typical characteristics of acute bacterial meningitis were developed, including superficial parenchymal damages, extensive focal hemorrhage with hemosiderin deposition **(E)**, chronic hemorrhage lesions **(F)**, edema in a perivascular region **(G)**, aggregation of microglial cells **(H)**, and micro-thrombosis **(I)**. Scale bar in **(A–I)** is 500 and 50 μm, respectively.

**Figure 3:**
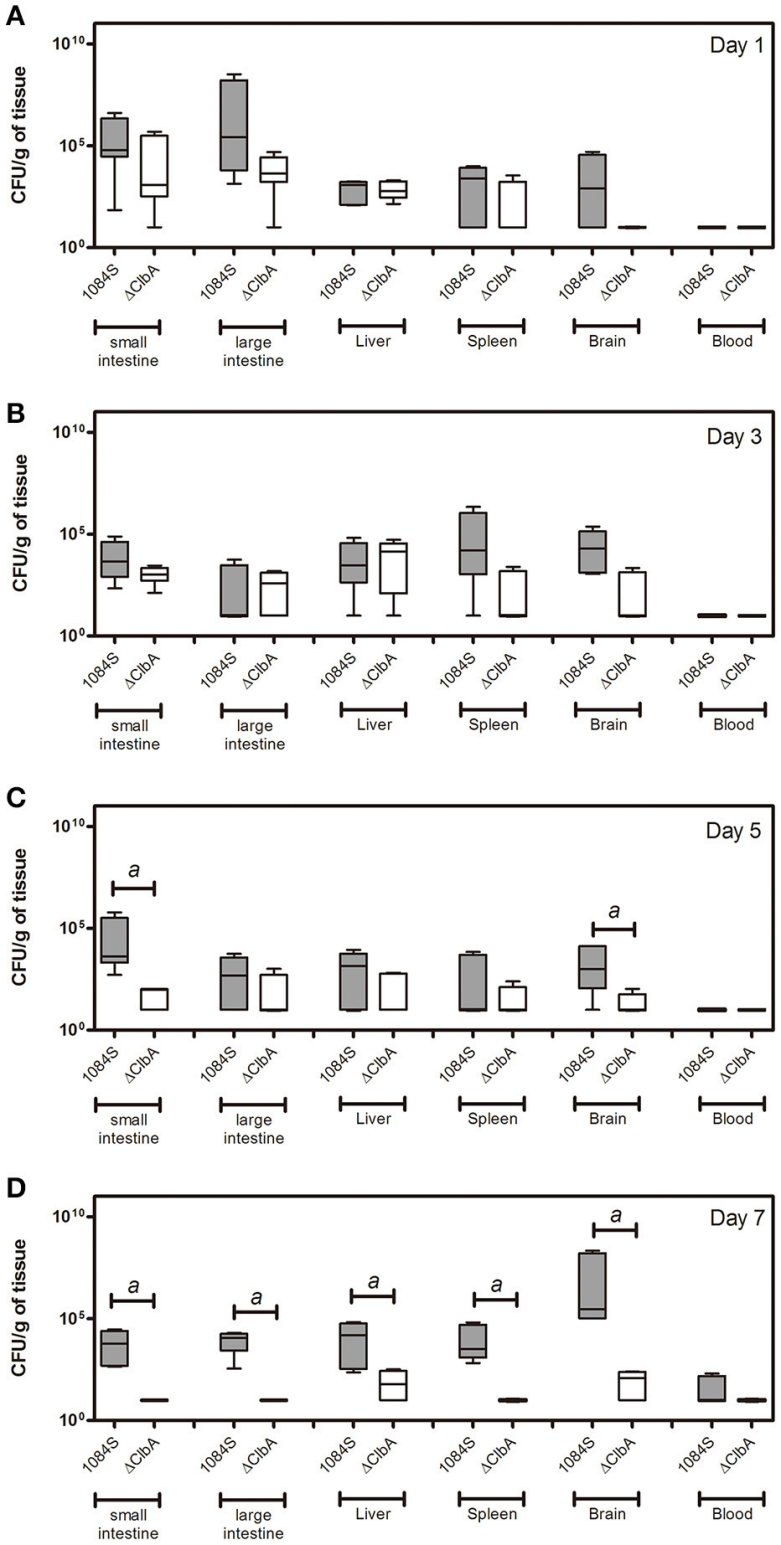
**Bacterial loads in various tissues of mice orogastrically infected with ***K. pneumoniae*** 1084S**. Groups of 8-wk old BALB/c male mice were orogastrically inoculated with 1 × 10^8^ CFU of *K. pneumoniae* 1084S or ΔClbA. Three to five mice were sacrificed at the day 1 **(A)**, day 3 **(B)**, day 5 **(C)**, and day 7 **(D)** post-inoculation. The mucosa of the small and large intestines, liver, spleen, brain, and blood were retrieved and homogenized for the determination of bacterial loads for the 1084S- (dark gray) and ΔClbA- (white) group. CFU per gram of tissues is presented in a box and whisker plot. The edges of each box are the 25th and 75th percentiles, and the middle is the median. Lower case letter “*a*” represents statistical significance of CFUg^−1^, *p* < 0.05 (one-tailed) determined by Mann-Whitney test, between 1084S and ΔClbA.

After translocation across the intestinal barrier, *K. pneumoniae* 1084S was prone to metastasize to the brain, leading to the development of meningitis in adult BALB/c mice. When compared to the control (Figure 2A), brains retrieved from these 1084S-infected mice showed typical signs of acute meningitis (Figure 2B). In subarachnoid space of brain, an enormous amount of *K. pneumoniae* and neutrophils accumulated (Figure 2D), induced superficial parenchymal damages, focal hemorrhage with hemosiderin deposition (Figure 2E), and chronic hemorrhage lesions (Figure 2F). Edema in a perivascular region (Figure 2G), aggregation of microglial cells (Figure 2H), and micro-thrombosis (Figure 2I) were also noted. To increase the survival rate of 1084S-infected mice, we reduced the inoculums to 1 × 10^8^ CFU and determined the bacterial loads in the intestines, liver, spleen, brain, and blood at the day 1, 3, 5, and 7 (Figure 3). Two of the five 1084S-inoculated mice developed systemic infections at the day 1 with the average number of bacterial loads in the liver, spleen, and brain as 1.7 × 10^3^, 8.3 × 10^3^, 3.6 × 10^4^ CFUg^−1^, respectively. At the same time point, in one of the inoculated mice, ΔClbA was detected in the liver (2.0 × 10^3^ CFUg^−1^) and spleen (3.4 × 10^3^ CFUg^−1^) but absent in the brain (Figure 3A). At the day 3, 80% of the mice (*n* = 5) were systemically infected with 1084S which had 1.9 × 10^4^ CFUg^−1^, 5.6 × 10^5^ CFUg^−1^, 7.3 × 10^4^ CFUg^−1^ in the liver, spleen, and brain, respectively. Although 80% of the mice (*n* = 5) were infected by ΔClbA in the liver (2.1 × 10^4^ CFUg^−1^) and spleen (1.6 × 10^3^ CFUg^−1^), only one of them had ΔClbA in the brain (1.3 × 10^3^ CFUg^−1^; Figure 3B). At the day 5, bacterial loads of ΔClbA in most of the tissues were decreased, while 1084S were maintained at the similar level as that of the day 3 (Figure 3C). One mouse of the 1084S group died at the day 6. All of the surviving mice developed meningitis at the day 7 with an average 1084S number of 5.0 × 10^7^ CFUg^−1^ in the brain (Figure 3D). More than half of the ΔClbA-inoculated mice successfully cleared ΔClbA at the day 7. Only two of the ΔClbA-inoculated mice carried bacterial loads in the liver (2.2 × 10^2^ CFUg^−1^) and brain (2.4 × 10^2^ CFUg^−1^). The use of lower infectious dose (1 × 10^8^ CFU) in this model revealed differences in host susceptibility to *K. pneumoniae* infections among individual mice. In general, the time required for most BALB/c mice to develop *K. pneumoniae* infections ranged from 1 to 5 days. Statistical differences between the 1084S and ΔClbA group were significant at the 7th day post-orogastric-inoculation.

**Figure 4:**
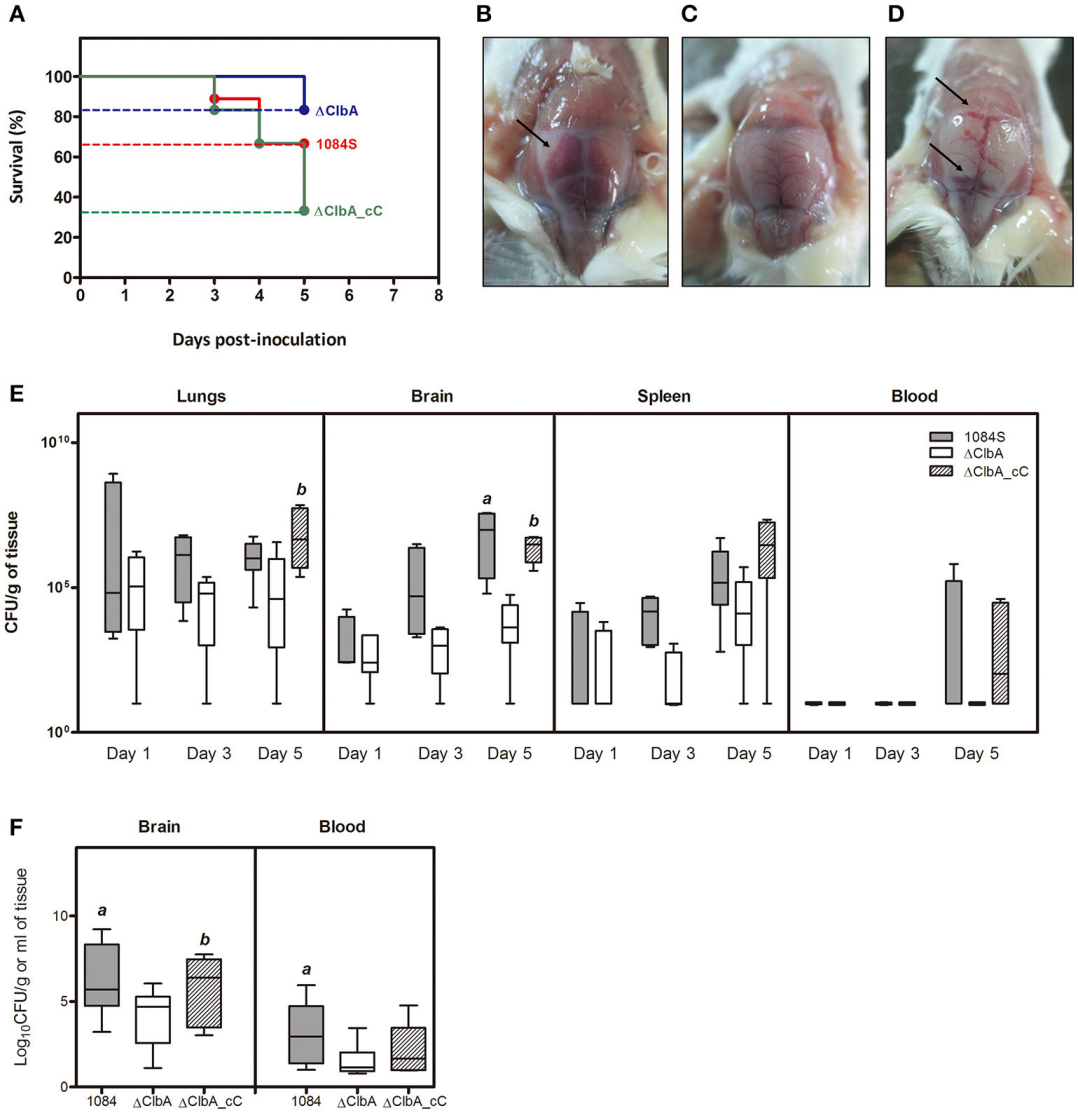
**Meningitis development following intranasal or intravenous inoculation of ***K. pneumoniae*** 1084S**. Groups of 8-wk old BALB/c male mice were intranasally inoculated with 1 × 10^6^ CFU of *K. pneumoniae* 1084S, ΔClbA, or ΔClbA_cC. **(A)** The 5-day survival rates of 1084S- (*n* = 9; red line), ΔClbA- (*n* = 6; blue line), or ΔClbA_cC-inoculated (*n* = 12; green line) mice determined by Kaplan-Meier analysis using Prism5 (GraphPad) are shown. At the 5th day post-inoculation, the infected mice which survived the experimental period were sacrificed. Representative images of the brain in the mice infected with *K. pneumoniae* 1084S **(B)**, ΔClbA **(C)**, or ΔClbA_cC **(D)** are shown. Arrows indicate subarachnoid hemorrhage. **(E)** Three to five mice were sacrificed at the day 1, 3, and 5 post-intranasal-inoculation. The lungs, brain, spleen, and blood were retrieved and homogenized for the determination of bacterial loads for the 1084S- (dark gray), ΔClbA- (white), and ΔClbA_cC- (slash) group. **(F)** Bacterial loads in the brain and blood were determined at 24 h after intravenous inoculation with 1 × 10^6^ CFU of *K. pneumoniae* 1084S (dark gray), ΔClbA (white), or ΔClbA_cC (slash). CFU per gram of tissues is presented in a box and whisker plot. The edges of each box are the 25th and 75th percentiles, and the middle is the median. Lower case letter “*a*” and “*b*” represents statistical significance of CFUg^−1^, *p* < 0.05 (one-tailed) determined by Mann-Whitney test, between 1084S/ΔClbA and ΔClbA/ΔClbA_cC, respectively.

We next used an intranasal instillation method to examine whether *K. pneumoniae* 1084 could induce meningitis via the respiratory route. To avoid the problem derived from the loss of pYC502, we constructed an in-*cis clbA* complement strain (named ΔClbA_cC), in which the full-length *clbA* gene was integrated into the *lacZ* locus of ΔClbA. Groups of 8-wk-old BALB/c mice were intranasally inoculated with 1 × 10^6^ CFU of 1084S, ΔClbA, or ΔClbA_cC. During the course of the experiment, several of the infected mice died due to severe sepsis. As shown in Figure 4A, the 5-day survival rate of the 1084S-, ΔClbA-, and ΔClbA_cC-inoculated mice were 66.7% (*n* = 9), 83.3% (*n* = 6), and 33.3% (*n* = 12), respectively. The surviving mice were sacrificed at the day 1, 3, and 5 post-inoculation to determine the bacterial loads in lungs, brain, spleen, and blood. Severe subarachnoid hemorrhage was observed in the brains which were harvested from the mice infected with 1084S (Figure 4B) or ΔClbA_cC (Figure 4D), as compared the ClbA group (Figure 4C). *K. pneumoniae* 1084S disseminated from the nasal tract, invaded the lungs, and spread to the brain and spleen. From day 1 to 5, no significant difference were observed in the lung bacterial loads between the 1084S and ΔClbA group (Figure 4E). However, all mice of the 1084S-inoculated group developed meningitis with increasing bacterial loads in the brain from day 1 (4.0 × 10^3^ CFUg^−1^) to day 5 (1.2 × 10^8^ CFUg^−1^). The deletion of *clbA* substantially impacted the capacity of *K. pneumoniae* 1084 to invade and replicate in the brain. However, through the intranasal route, ΔClbA was still able to maintain its numbers in the lungs and disseminated to the spleen (Figure 4E). To examine whether colibactin participated in other steps during meningitis development, we directly injected 1 × 10^6^ CFU of 1084S, ΔClbA, or ΔClbA_cC into the blood circulation of BALB/c mice via the tail vein to bypass the mucosal barrier. At 24 h post-inoculation, one-fourth of the 1084S-inoculated mice which carried brain bacterial loads over 10^8^ CFUg^−1^ had developed acute bacterial meningitis, whereas none of the ΔClbA-inoculated mice exhibited prominent signs of meningitis. The bacterial counts in the brain of ΔClbA-inoculated mice were significantly less than that of the 1084S group (Figure 4F). The defect of ΔClbA on the *K. pneumoniae* access to the brain was partly attributed to the increase in bacterial clearance of ΔClbA in the blood, as shown in Figure 4F that the blood burden of ΔClbA was ~20-fold lower than that of 1084S. In-*cis*-complementation of *clbA* restored the ability of ΔClbA to induce meningitis (ΔClbA_cC in Figure 4). These results suggested that the production of colibactin was required for the meningeal tropism of *K. pneumoniae* 1084S following the translocation across a respiratory barrier and was also involved in providing *K. pneumoniae* resistance to bacterial clearance in bloodstream.

### Inflammatory responses to *K. pneumoniae*-induced acute meningitis in BALB/c mice

**Figure 5:**
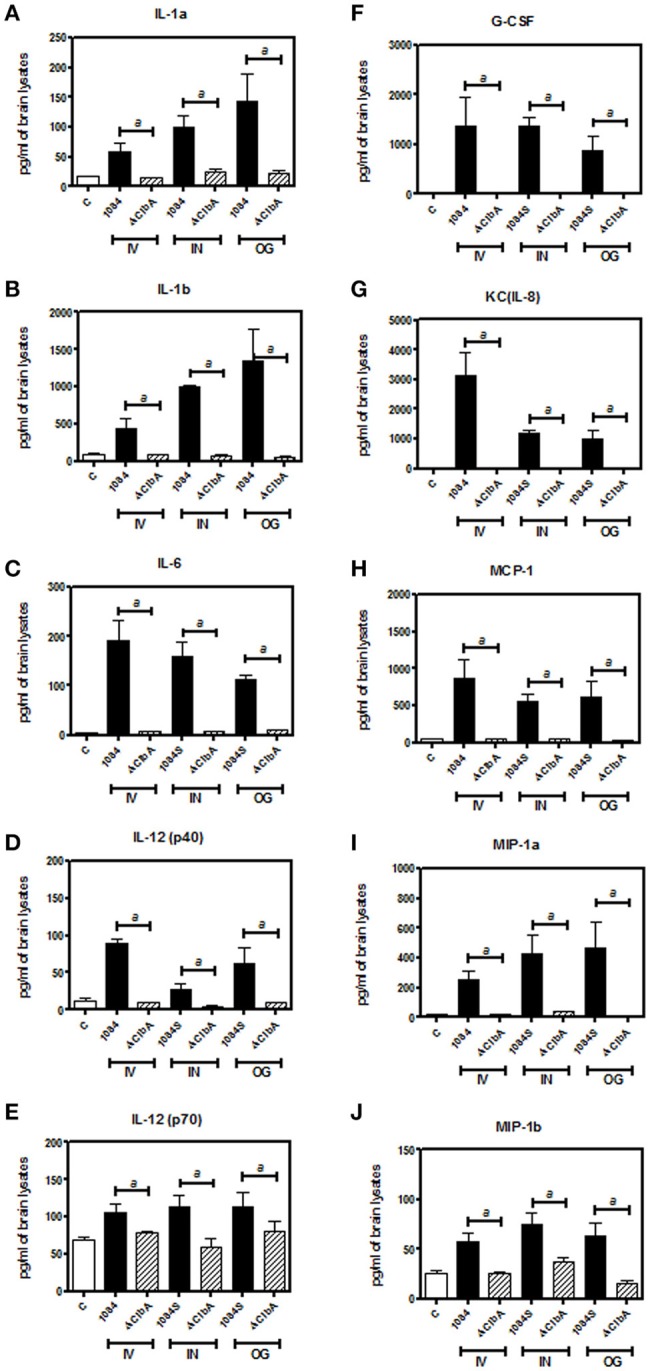
**Inflammatory responses upon ***K. pneumoniae***-induced meningitis**. Brain tissues retrieved from three of the 1084S- and ΔClbA-inoculated mice which had the highest brain CFU at 24 h post-intravenous-inoculation (IV), 5 days post-intranasal-inoculation (IN), and 7 days post-orogastric-inoculation (OG), were homogenized with lysis buffer, protein-concentration determined, and subjected to quantification of 23 cytokines and chemokines. Inflammatory cytokines, IL-1a **(A)**, IL-1b **(B)**, IL-6 **(C)**, IL-12 (p40) **(D)**, and IL-12 (p70) **(E)**, G-CSF **(F)**, and chemokines, KC **(G)**, MCP-1 **(H)**, MIP-1a **(I)**, and MIP-1b **(J)**, which were significantly induced upon *K. pneumoniae* 1084S infections are shown. Data are expressed as mean ± SEM. “*a*” represents a significant increase in the *K. pneumoniae* 1084S-infected group (black bars) in comparison to the ΔClbA group (slash bars), determined by Student's *t*-test (one-tailed; *p* < 0.05).

**Figure 6:**
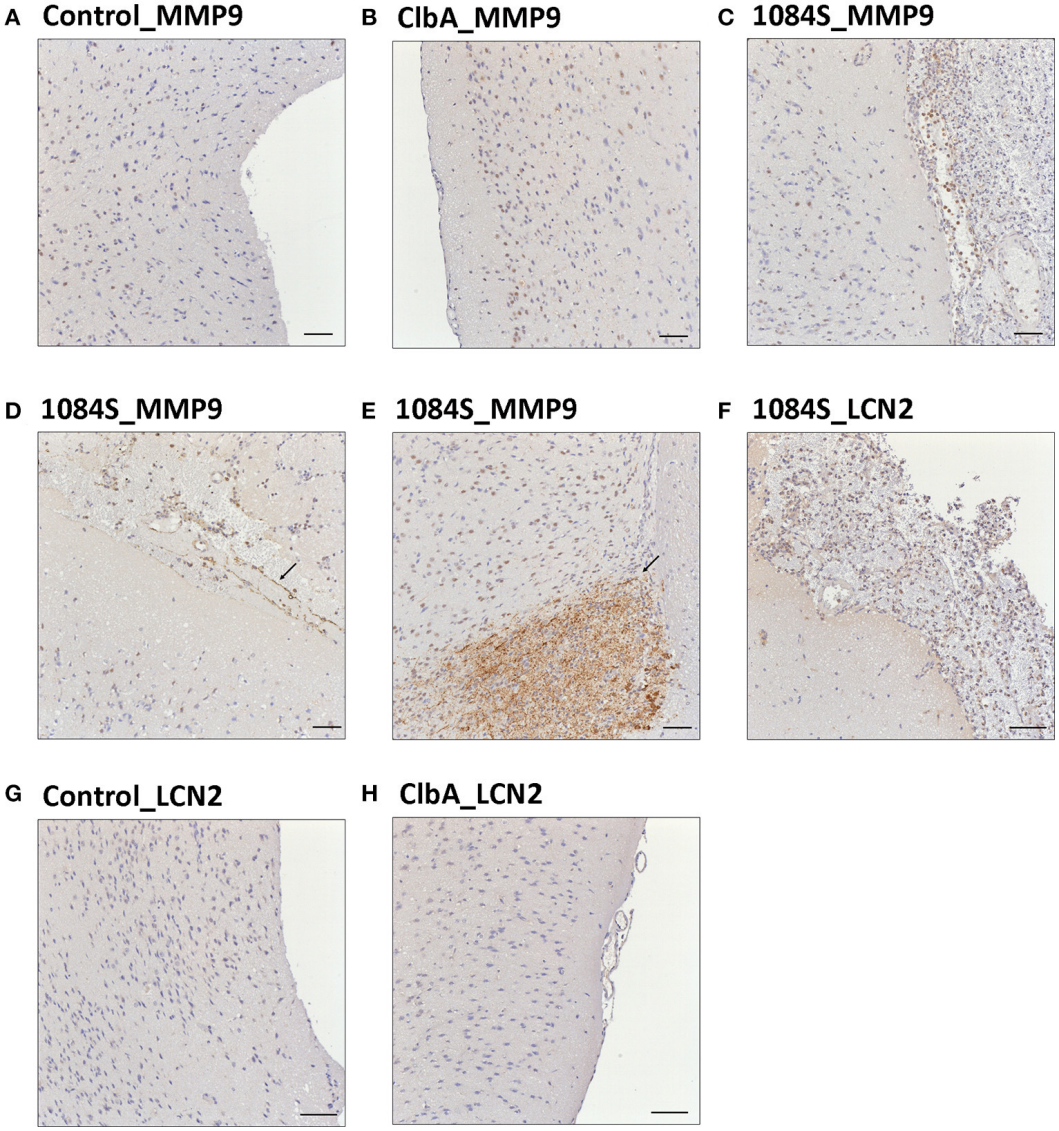
**Immunohistochemical analysis of MMP-9 and LCN-2 expression in the brain upon ***K. pneumoniae*** 1084S infections**. Representative images of brain sections retrieved from the control mice **(A,G)**, the ΔClbA-inoculated mice **(B,H)**, and the 1084-inoculated mice which developed meningitis **(C–F)** at the 7th day post-orogastric-inoculation are shown. Brown staining (arrows) indicates a positive reaction with the respective antibody against MMP-9 **(A–E)** and LCN-2 **(F–H)**. Scale bar: 50 μm.

Meningitis-induced brain dysfunction is partly attributed to the enhanced release of host inflammatory cytokines, chemokines, proteolytic enzymes, and oxidants. We next examined whether the infection of *K. pneumoniae* 1084S caused overwhelming inflammation leading to the brain damages. Three mice which had the highest brain CFU at 24 h post-intravenous-inoculation, 5 days post-intranasal-inoculation, and 7 days post-orogastric-inoculation, were selected from each group. Levels of 23 cytokines and chemokines in the brains which were retrieved from the mice with a profound brain burden of *K. pneumoniae* were determined. Compared to ΔClbA and the control group, production of inflammatory cytokines IL-1a, IL-1b, IL-6, IL-12, and G-CSF, and chemokines KC (IL-8), MCP-1 (CCL2), MIP-1a (CCL3), and MIP-1b (CCL4), was significantly increased upon *K. pneumoniae* 1084S infections in the brain (Figure 5). Granulocytes, mainly neutrophils, could be recruited from circulation by the elevated levels of KC, MIP-1a, and MIP-1b, accumulated in the subarachnoid space and resulted in acute neutrophilic inflammation. Consistently, at the 7th day post-orogastric-inoculation, we observed that vigorous infiltration of neutrophils occurred concomitantly with bacterial accumulation in subarachnoid space of brain (Figure 2D). In addition to the incitement of inflammatory cytokines and chemokines, compared to the control (Figure 6A) and ΔClbA group (Figure 6B), matrix metalloproteinase (MMP)-9 expression was increasingly detected in the neutrophil influx (Figure 6C), in the endothelial lining of blood vessels (Figure 6D), and in the brain parenchyma (Figure 6E) of the 1084S-infected mice. Moreover, a subset of infiltrating neutrophils was LCN (lipocalin)-2 positive (Figure 6F), which was absent in control (Figure 6G) and ΔClbA group (Figure 6H). MMP-9 and LCN-2 released from activated neutrophils might contribute to the leakage of the blood-brain barrier and subsequent brain edema (Shigemori et al., 2006).

### *pks*^+^ K1 CC23 *K. pneumoniae* induced DNA damages and cell death in the brain

**Figure 7:**
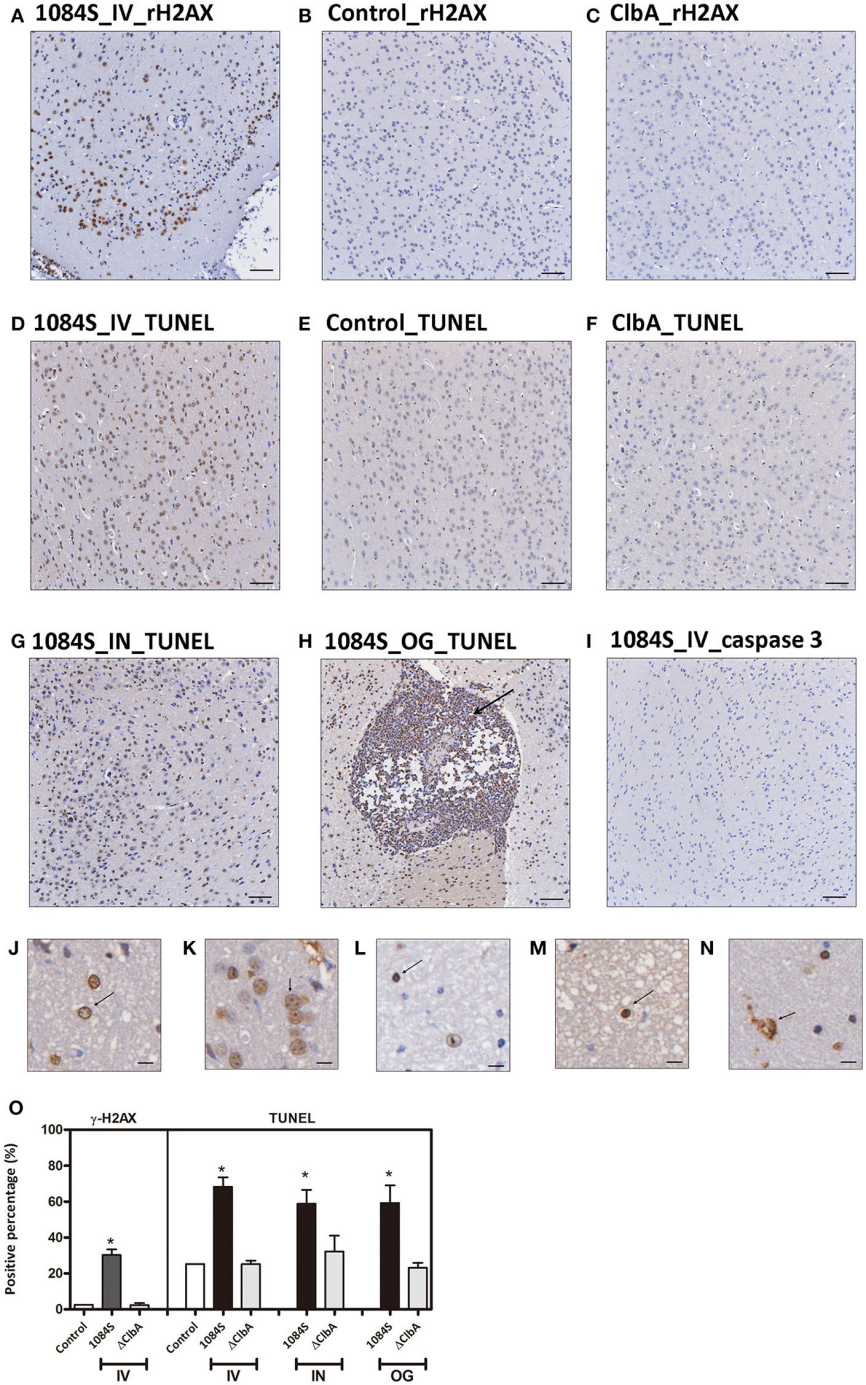
**DNA damages and cell death in response to ***K. pneumoniae*** 1084S meningitis. (A–C)** DNA damages. Brain sections of the 1084-inoculated mice which developed meningitis at 24 h post-intravenous-inoculation **(A)** and sections of the control **(B)**, and ΔClbA-inoculated mice **(C)** were stained for γ-H2AX. **(D–I)** Cell death. Brain sections retrieved from the 1084S-inoculated mice which developed meningitis at 24 h post-intravenous-inoculation (IV) **(D)**, at the 5th day post-intranasal-inoculation (IN) **(G)**, and at the 7th day post-orogastric-inoculation (OG) **(H)**, and the corresponding control **(E)** and the ΔClbA-inoculated mice **(F)** were subjected to TUNEL assay **(D–H)** and the detection of cleaved caspase 3 **(I)**. Representative images are shown. Brown staining indicates a positive reaction with the respective antibody. Scale bar: 50 μm. **(J–N)** Nuclear alterations of the brain cells in response to *K. pneumoniae* 1084S meningitis. Karyorrhexis (nuclear fragmentation), pyknosis (nuclear shrinkage), and karyolysis (nuclear fading) are presented in **(J–N)**, respectively. Scale bar: 5 μm. **(O)** Positive cells for respective antibodies in brain sections were quantified using the HistoQuest software of TissueFAXS plus system (TissueGnostics GmbH). Data represent means ± SEM from three mice per group. An asterisk (^*^) represents statistical significance, *p* < 0.05 (one-tailed) determined by Student's *t*-test, between 1084S and ΔClbA.

Through the intravenous delivery, ΔClbA reached the brain with bacterial loads ranging from 10^2^ to 10^6^ (Figure 4F), which was ~1,000-fold lower than the burden of 1084S. Based on the neuropathological analysis, only mild inflammation was detected in the ΔClbA-inoculated mice. Consistent with this result, the induction of inflammatory cytokines (Figure 5) and the recruitment of MMP-9- and LCN-2-positive neutrophils (Figures 6B,H) were unobvious in the ΔClbA group. Given *K. pneumoniae* 1084 is a colibactin-producing K1 CC23 strain, we next examined whether the infection of 1084S caused DNA damages in the brain by detecting phosphorylation of histone H2AX, an indicator of double-strand DNA breaks. At 24 h after intravenous inoculation of *K. pneumoniae* 1084S, ~30% of brain parenchymal cells had undergone DNA damages, showing positive signals of γ-H2AX in their nuclei (Figure 7A). As γ-H2AX-positive cells were hardly detected in control (Figure 7B) and ΔClbA (Figure 7C) group, an involvement of colibactin was suggested in the induction of DNA damages in the 1084S-infected brain. Besides, extensive cell death in the brain parenchyma was noted. As shown in Figure 7D, TUNEL-positive cells were significantly increased in the 1084S-infected brain when compared to the control (Figure 7E) and ΔClbA (Figure 7F) group. Through the other routes of inoculation, *K. pneumoniae* 1084S also elicited similar levels of cell death in the brain (Figure 7G, 5 days post-intranasal; Figure 7H, 7 days post-orogastric). Besides, neutrophil cell death was concomitantly observed (Figure 7H, arrow). In response to *K. pneumoniae* 1084S-induced meningitis, various alterations in the nucleus of brain cells occurred. Karyorrhexis (nuclear fragmentation; Figures 7J,K), pyknosis (nuclear shrinkage; Figures 7L,M), and karyolysis (nuclear fading; Figure 7N) are noted on the TUNEL-positive cells in most of the brain sections with severe *K. pneumoniae* 1084S-induced meningitis. The absence of cleaved caspase 3 signals suggested that the extensive cell death elicited by *K. pneumoniae* 1084S was activated through a caspase 3-independent pathway (Figure 7I).

## Discussion

Despite the advance of clinical techniques and the use of new antibiotics, bacterial meningitis remains the leading cause of death and long-term neurological sequelae. *K. pneumoniae* is a common etiological organism for adult bacterial meningitis, particularly in the diabetic population, with an incidence ranging from 25.5 to 31.7% in Taiwan (Huang et al., 2002; Chang et al., 2008). However, relatively few literature reported experimental models of *K. pneumoniae* meningitis (Wen et al., 2007; Barichello et al., 2014). Barichello et al. (2014) delivered *K. pneumoniae* directly through an intracisternal route to induce meningitis and demonstrated that the increase of proinflammatory responses caused memory impairments in the inoculated Wistar rats (Barichello et al., 2014). Except for post-neurosurgical meningitis, the majority of *K. pneumoniae* meningitis cases involve an intestinal/respiratory portal of entry, access to circulation, and penetration across the brain barrier. To elucidate the molecular mechanism underlying the key pathogenic steps, we aimed in this study to establish a mouse model that followed the natural pathway to induce *K. pneumoniae* meningitis.

*K. pneumoniae* 1084 is a colibactin-producing K1 CC23 strain (Lai et al., 2014). Compared to other common K1 CC23 *K. pneumoniae* strains that cause the pyogenic liver abscess, 1084 acquires a gene cluster ~100% identical to the *pks* island (Nougayrede et al., 2006) which encodes an NRPS-PKS complex for the biosynthesis of colibactin. Similar to what have been demonstrated in *pks*^+^ *E. coli*, deletion of *clbA* abolished the production of colibactin and substantially attenuated the genotoxicity of *K. pneumoniae* 1084 to mammalian cells (Lai et al., 2014). In addition to the genotoxicity, *K. pneumoniae* 1084 exhibited meningeal tropism in adult BALB/c mice. After the reduction of gut microbiota by the addition of streptomycin in drinking water, the orogastrically inoculated *K. pneumoniae* 1084S (a streptomycin-resistant variant of 1084; 10^9^ CFU) established intestinal colonization, rapidly disseminated, and induced meningitis in all of the inoculated mice within 7 days. The meningeal tropism of *K. pneumoniae* 1084 was unobserved in *K. pneumoniae* CG43, a strain that we have used for the liver abscess model in BALB/c mice (Tu et al., 2009). After colonizing the intestines, CG43 could also invade extraintestinally and develop systemic infections, but hardly induced meningitis at the same time scale. CG43 is a K2 ST86 strain with hypervirulence but is colibactin negative. We next examined the extent of involvement of colibactin in the meningeal tropism of *K. pneumoniae* 1084. Although ΔClbA replicated in the intestines comparably to its parental strain 1084S (Figure 1A), its adherence to the mucosa of small and large intestines was significantly affected by the deletion of *clbA* (Figure 1B). This result was in line with the observation that the presence of colibactin correlated to the capacity of *E. coli* strains of phylogenetic group B2 to maintain long-term colonization in the intestines (Nowrouzian and Oswald, 2012).

Intestinal colonization is a prerequisite for *K. pneumoniae* to develop systemic infections (Tu et al., 2009; Fung et al., 2012). The deletion of *clbA* affected *K. pneumoniae* colonization of the intestinal mucosa and the subsequent translocation into extraintestinal tissues. This result suggested a role of colibactin in the intestinal translocation of *K. pneumoniae* 1084S. The production of colibactin did not cause intestinal injuries (Supplementary Result; Figure S1). However, the largest area of the small intestines retrieved from the 1084S group was found to have averagely enlarged submucosal space with slight hyperemia (Figure S1E) compared to the ΔClbA group (Figure S1F). This result suggested that the production of colibactin might promote inflammation in the intestinal mucosa. Comparative RNA-seq analyses of the mucosa demonstrated that subsets of inflammation-associated genes were significantly upregulated in the *K. pneumoniae* 1084S inoculated mice when compared to the ΔClbA group (Supplementary Results; Figure S2). CCL17, CCL8, and MMP9 were significantly upregulated (>10 Log_2_) in response to the colibactin-producing *K. pneumoniae*. The chemokine CCL17, expressed by conventional DCs, has an autocrine effect on DCs that promotes production of inflammatory cytokines and activation of Th1 and Th17 cells and reduces expansion of Treg cells (Heiseke et al., 2012). MMP9 derived from polymorphonuclear neutrophils has been demonstrated to cause intestinal barrier dysfunction and promote bacterial translocation (Mikami et al., 2009). Our preliminary result suggested that the production of colibactin might enhance intestinal inflammation through a yet-unknown mechanism. The inflammatory status might create a portal of entry for *K. pneumoniae* to get access to the brain.

In the intranasal model, ΔClbA, as its parental strain, traveled down from nasal passage into the lower airway, maintained its burden at the wild-type level from day 1 to 5 in the lungs, and disseminated to the spleen (Figure 4E). However, the number of ΔClbA in the blood was significantly reduced (Figures 4E,F). Increased clearance of ΔClbA in the blood affected its invasion to the brain. The blood circulation is traditionally considered the sole entry to the central nervous system (CNS) for neuropathogens. However, as revealed by drug delivery studies, direct transport of particles may occur from the nasal cavity to the CSF (cerebrospinal fluid; Mistry et al., 2009). Also, the newly discovery of the CNS glymphatic (Yang L. et al., 2013) and lymphatic system (Louveau et al., 2015) indicates that the complexity of the nervous system is beyond the traditional thinking. We cannot exclude the possibility that *K. pneumoniae* 1084 may take advantages of other passages rather than the blood to gain access to CNS, which might require the involvement of colibactin.

ClbA, a 4′-phosphopantetheinyl transferase (PPTase), is required for the biosynthesis of colibactin. In *E. coli*, ClbA is also involved in the production of PPTase-dependent siderophores (salmochelin, enterobactin, and yersiniabactin; Martin et al., 2013). However, the deletion of *clbA* did not influence the production of siderophores in *K. pneumoniae* 1084, as measured by SideroTec assay (Emergen Bio) that the average quantity of siderophores produced by1084S and ΔClbA was 82 and 85 μg/ml, respectively. The lack of impact of ClbA on the production of siderophores in *K. pneumoniae* 1084 may be because the dominant siderophore produced by this strain is aerobactin. Its biosynthesis is encoded by *iucABCD* operon which is independent of PPTase. The deletion of *iucA*, which abolished the production of aerobactin, attenuated *K. pneumoniae* virulence *in vivo*. Salmochelin, enterobactin, and yersiniabactin are dispensable for the growth and survival of hypervirulent *K. pneumoniae* strains *ex vivo* and *in vivo* (Russo et al., 2015). The influence of *clbA* deletion on the meningeal tropism of *K. pneumoniae* 1084 reported here is mostly irrelevant to siderophores and can be primarily attributed to the production of colibactin. Blockage of colibactin production affecting *K. pneumoniae* virulence was validated by analyzing the *clbP* deletion mutant. The *clbP* gene encodes a D-amino peptidase involved in the maturation of colibactin. Precolibactin is transported into the periplasm and cleaved by ClbP to release the mature product (Dubois et al., 2011; Cougnoux et al., 2012). The deletion of *clbP* has been demonstrated to abolish the production of the biological effect of colibactin in *E. coli* (McCarthy et al., 2015; Cougnoux et al., 2016; Trautman et al., 2017). Similar to ΔClbA, ΔClbP also exhibited attenuated virulence in the three meningitis mouse models (Supplementary Results; Figure S3). These results collectively suggested that the production of colibactin rendered *K. pneumoniae* 1084 hypervirulence and that was critical for the development of meningitis.

Neurological deficits were noted in some of the BALB/c mice suffered from *K. pneumoniae* 1084 meningitis. These mice exhibited severe defects on balance, as manifested by exaggerated lean to one side of the body with longitudinal spinning and rolling (Supplementary Video). Both the host and bacterial factors can contribute to brain deficits. Besides typical characteristics of bacterial meningitis (Figure 2), severe inflammation of the brain parenchyma (Figure 5) was evoked upon *K. pneumoniae* infections. Production of inflammatory cytokines IL-1a, IL-1b, IL-6, IL-12, and G-CSF, and chemokines KC (IL-8), MCP-1 (CCL2), MIP-1a (CCL3), and MIP-1b (CCL4) significantly increased in the brain of the infected mice (Figure 5). Upon the exposure to *K. pneumoniae* in the subarachnoid space, brain immune cells, such as astrocytes and microglia, were likely to be activated and released inflammatory cytokines and chemokines. IL-1b was found in the CSF of human patients with bacterial meningitis (Ostergaard et al., 2004) and was the initial cytokine produced upon the induction of pneumococcal meningitis in a rat model (Barichello et al., 2010). In response to IL-1b, IL-6 was elevated and predominantly stimulated the acute phase protein synthesis, fever response (Gruol and Nelson, 1997), and contributed to the motor coordination deficit (Yang S. H. et al., 2013). The chemoattractant properties of the chemokines, such as G-CSF, KC (IL-8), MIP-1a, and MIP-1b observed in our *K. pneumoniae* experimental meningitis model, recruited granulocytes, particularly neutrophils, to the inflammatory site. Accumulation of massive neutrophils consequently led to acute neutrophilic inflammation. Although *K. pneumoniae* could be engulfed and killed by the recruited neutrophils, the release of cell-damaging intermediates simultaneously elicited brain tissue damages. Matrix metalloproteases (MMPs) are peptides playing an important role in brain injury during infection of the central nervous system. An elevated level of MMP9 could contribute to the leakage of the blood-brain barrier and inflammatory infiltration (Sellner and Leib, 2006), which exaggerated inflammatory response and neuronal injury. Lipocalin 2 (LCN2) is an acute phase protein, whose concentrations in CSF in human patients with acute bacterial meningitis are highly increased (Guiddir et al., 2014). Multifaced roles played by LCN2 include modulating activation of microglia, astrocytes, and endothelial cells through M1 polarization and TLR signaling pathways, inducing neuronal cell death (Bi et al., 2013), and sequestering siderophores (mainly yersiniabactin) to inhibit bacterial survival (Bachman et al., 2009, 2011). The overwhelming production of proinflammatory cytokines, accumulation of neutrophils, and elevation of MMP9 and LCN2 collectively exacerbated the brain damages induced upon *K. pneumoniae* meningitis.

*K. pneumoniae* meningitis resembled those caused by other common bacterial neuropathogens (Roos, 2000) in the histopathological and inflammatory characteristics. Moreover, this colibactin-producing *K. pneumoniae* strain exerted genotoxicity in the brain. At 24 h after an intravenous inoculation of *K. pneumoniae* 1084S, exceeding 30% of brain parenchymal cells showed positive signals for γH2AX, an indicator of DNA double strand breaks (Figures 7A,O). Massive extent of cell death was concurrently detected by TUNEL assay (Figures 7D,G,H,O). Various alterations of the nucleus were observed in the TUNEL-positive brain cells, including karyorrhexis (nuclear fragmentation; Figures 7J,K), pyknosis (nuclear shrinkage; Figures 7L,M), and karyolysis (nuclear fading; Figure 7N). Karyolysis indicates necrotic cell death, but pyknosis and karyorrhexis are found in both necrosis and apoptosis. Further, investigation is needed to elucidate whether the TUNEL-positive brain cells are in different stages of necrosis or undergo different forms of cell death. The absence of cleaved caspase 3 shown in Figure 7I suggested a caspase 3-independent pathway activated by *K. pneumoniae*-induced meningitis that elicited the extensive cell death. The caspase 3-independent cell death has been demonstrated in pneumococcal meningitis. The release of pneumolysin and H_2_O_2_ from *S. pneumoniae* D39 elevated the level of intracellular Ca^2+^ and triggered the translocation of AIF (apoptosis-inducing factor; Braun et al., 2002). Human survivors of bacterial meningitis frequently suffer from long-term neurologic sequelae, such as seizures and hearing/vision loss. The cell death induced by meningeal pathogens contributes to the damages of brain tissues. Further, studies are needed to elucidate the mechanism of cell death induced by *K. pneumoniae* meningitis and the involvement of colibactin and the related genotoxicity in this process.

Despite all experimental animal models have limitations to completely mimic human disease; unquestionably, they are still a valuable tool to clarify the pathogenesis mechanism. *K. pneumoniae* infections have multifaced manifestations in human. Meningitis is one of the most severe diseases caused by hypervirulent *K. pneumoniae* strains which carry a particular subset of meningeal factors. Research into its pathogenesis mechanism, however, has been so far impeded due to the lack of a physiologically relevant model. For the first time, we used the hypervirulent *pks*^+^ K1 CC23 *K. pneumoniae* strain 1084 to successfully induce meningitis in adult BALB/c mice through the orogastric and intranasal routes. The meningeal tropism of this *K. pneumoniae* strain allowed the development of meningitis prior to the induction of septic shock in the majority of infected mice. The meningitis model established in this study is suitable for in-depth analyses of the different pathogenic steps occurring from colonization to disease in CNS. The carriage of *pks* island, which is responsible for the biosynthesis of colibactin, is the most profound characteristic of *K. pneumoniae* 1084. In the meningitis mouse model established here, we demonstrated that the blockage of the colibactin production, through the deletion of *clbA* or *clbP*, substantially hindered *K. pneumoniae* hypervirulence in the key pathogenic steps toward the development of meningitis. Owing to the fact that ΔClbA/ΔClbP was significantly attenuated, the loads of ΔClbA/ΔClbP in the brain cannot reach the threshold to induce meningitis. It is not applicable to determine the direct effect of colibactin on the brain tissue. If colibactin can be purified in the near future, we could inject it directly into the brain and analyze its pathological effects on the brain tissue. At present, only few meningitis mouse models have been established with the inoculation of neuropathogens via the natural pathways. The *K. pneumoniae* meningitis mouse model in this study is valuable that it can be further applied to identify both the bacterial and host factors participating in the development of this life-threatening disease. Through elucidating the molecular mechanism underlying the bacterial pathogenesis, we will have the chance to translate the insights into the clinical practice.

## Author contributions

ML, YC, MC, and YL performed the analysis and interpretation of data. ML, MC, and YL drafted the manuscript. PH and YH performed the animal studies. YC, YW, CC, CLL, and CTL made substantial contributions to conception and design and revised the manuscript critically for important intellectual content. All authors read and approved the final manuscript.

## Funding

This work was supported by the Ministry of Science and Technology of Taiwan [MOST 104-2320-B-040-021-] and [MOST 103-2314-B-040-020-MY3] and also in part by innovative research grants of National Health Research Institutes [NHRI-EX104-10327BI] and Chung Shan Medical University Hospital [CSH-2016-C-017] and [CSH-2014-C-030]. The funders had no role in study design, data collection, and analysis, decision to publish, or preparation of the manuscript.

### Conflict of interest statement

The authors declare that the research was conducted in the absence of any commercial or financial relationships that could be construed as a potential conflict of interest.
