# Supplementary material for: Colibactin Contributes to the Hypervirulence of pks+ K1 CC23 Klebsiella pneumoniae in Mouse Meningitis Infections
Source: Front Cell Infect Microbiol. 2017 Mar 31;7:103. doi: 10.3389/fcimb.2017.00103 (PMC5374149; doi:10.3389/fcimb.2017.00103)
Supplement: Supplementary file 2 [file Presentation1.pdf]

Supplementary results

Examination of intestinal mucosa in response to orogastric inoculation of *K. pneumoniae* 1084S and ΔClbA. Intestinal colonization is a prerequisite for *K. pneumoniae* to develop systemic infections (Tu et al., 2009;Fung et al., 2012). Through the orogastric route, *K. pneumoniae* 1084S established its population in the small intestinal mucosa during the seven days post-inoculation, whereas ΔClbA could not be maintained, with decreasing loads from the 5th day (Fig. 3). To examine whether the production of colibactin by *K. pneumoniae* 1084S induced tissue damages to assist the translocation across an intestinal barrier, we collected the whole small intestines from the mice which were orogastrically inoculated with 1×10^8 CFU of 1084S or ΔClbA at the 3rd day post-inoculation. Histological examination of the hematoxylin and eosin (H/E) stained sections revealed no significant mucosal damages in the 1084S-infected intestines when compared to the control and that of ΔClbA group (Fig. S1). However, the largest area of the small intestines retrieved from the 1084S group was found to have averagely enlarged submucosal space with slight hyperemia (Fig. S1E) as compared to the ΔClbA group (Fig. S1F). The size of Peyer's patches in the 1084S group (Fig S1H) was comparable to that in the ΔClbA group (Fig. S1I). However, comparative RNA-seq analyses of the mucosa revealed that the production of colibactin by *K. pneumoniae* 1084 promoted intestinal inflammation, which might consequently assist bacterial translocation. Briefly, intestinal mucosa was collected from *K. pneumoniae* 1084S- and ΔClbA-inoculated mice at the day 3 for comparative RNA-Seq analyses (Fig. S2A). Clean reads were obtained from the raw reads by removing the adaptor sequences, reads with >5% ambiguous bases, and low-quality reads. The clean reads were then aligned to the mouse genome. The differently expressed genes were screened out using the following criteria: 1) fold change (FC) >2 for up- or downregulation and 2) false discovery rate (FDR) <0.05. Genes with FC >2 for both up- or downregulation and FDR <0.05 were identified as significantly differently expressed. Compared to the intestinal mucosa infected with ΔClbA, a total of 526 LncRNAs displayed differential expression in the colibactin-producing *K. pneumoniae* infected mucosa tissue, including 384 upregulated LncRNAs and 142 downregulated LncRNAs. To elucidate the biological implication of unique genes with a role in response to *K. pneumoniae* 1084S colonization, we included all differently expressed mRNAs for GO (Gene ontology) analysis (Fig. S2B). In the GO biological process analysis, the most enriched was "extracellular region". The majority of genes were related to the extracellular region in the cellular component analysis and calcium ion binding in the molecular function. In the KEGG (Kyoto Encyclopedia of Genes and Genomes

database) pathway analysis, the dysregulated mRNAs were found to be enriched in 28 pathways (Fig. S2C). Fold of activation of genes categorized in the chemokine signaling pathway and leukocyte transendothelial migration is presented (Fig. S2D). CCL17, CCL8, and MMP9 were significantly upregulated (> 10 Log_2) in response to the colibactin-producing *K. pneumoniae*. The chemokine CCL17, expressed by conventional DCs, has been demonstrated to be required for induction of intestinal inflammation in mice and has an autocrine effect on DCs that promotes production of inflammatory cytokines and activation of Th1 and Th17 cells and reduces expansion of Treg cells (Heiseke et al., 2012). The chemokine CCL8, secreted by CD169 (+) macrophage in response to mucosal instability, has been demonstrated to serve as an alert signal for the collapse of mucosal barrier defense (Asano et al., 2015). In rat acute pancreatitis model, MMP9 derived from polymorphonuclear neutrophils has been demonstrated to cause intestinal barrier dysfunction and promote bacterial translocation (Mikami et al., 2009). Our preliminary result suggested that the production of colibactin might induce intestinal inflammation through a yet-unknown mechanism. The inflammatory status in the intestinal mucosa may facilitate tight junction disruption, intestinal permeability, and transendothelial migration of leukocytes and that provides *K. pneumoniae* a portal of entry into extraintestinal tissues.

Generation of the *clbP* deletion mutant in *K. pneumoniae* 1084S. A 1506-bp region spanning the coding sequence of *clbP* was deleted in *K. pneumoniae* 1084S by using an allelic exchange technique. In general, 1,200-bp DNA fragments flanking the region to be deleted were amplified with specific primer sets, p540 (TCTAGATCATTTGGTTCTGCAAAACTGGT)/p541 (GGTACCTATTGTCATCCTGTGAACACCT) and p542 (GGTACCTGGGACGATGAGTAATATCAGT)/p543 (GAGCTCTGAATCATACGACCTCGGGT) and the amplified DNA fragments were cloned into pKAS46, a suicide vector containing *rpsL*, which allows positive selection for vector loss using streptomycin (Skorupski and Taylor, 1996). To facilitate the positive selection of deletion mutants, a chloramphenicol-resistant cassette which was amplified from pACYC184, cloned into the insert on pKAS46 to replace the deletion region. The resulting construct pYC519 in *E. coli* S17-1 λ *pir* was subsequently mobilized to *K. pneumoniae* 1084S via conjugation. Kanamycin-resistant transconjugants were selected, propagated in LB, and then subjected to streptomycin selection (500 μg/ml). After the occurrence of double cross-over, colonies showing resistance to chloramphenicol and susceptibility to kanamycin were isolated. After PCR verification, the mutant with *clbP* deletion was named ΔClbP.

Deletion of *clbP* attenuated *K. pneumoniae* 1084 virulence in mouse meningitis model. While the *clbA* gene encodes a phosphopantetheinyl transferase required for colibactin synthesis, the *clbP* gene encodes a D-amino peptidase involved in the maturation of colibactin. Precolibactin is transported into the periplasm and cleaved by ClbP to release the mature product (Dubois et al., 2011;Cougnoux et al., 2012). The deletion of *clbP* has been demonstrated to abolish the production of the biological effect of colibactin in *E. coli* (McCarthy et al., 2015;Cougnoux et al., 2016;Trautman et al., 2017). To examine whether the defects of ΔClbA on the development of meningitis were attributed to the lack of colibactin, we determined the virulence of ΔClbP in our meningitis mouse models. Through the orogastric route, 200 μl of bacterial suspension containing 1×10^8 CFU of ΔClbP were inoculated into six 8-wk-old BALB/c male mice. At the 7th day post-inoculation, all of the mice were sacrificed. The mucosa of small and large intestines, liver, spleen, brain, and blood were retrieved, homogenized, and subjected to the enumeration of ΔClbP. Similar to ΔClbA, ΔClbP was able to propagate in the intestinal lumen, as shown in supplementary Fig. 3A that the fecal shedding of ΔClbP was not significantly affected. However, when compared to 1084S, the loads of ΔClbP significantly decreased in the intestinal mucosa and in most of the extraintestinal organs, including the brain. The defect of ΔClbP on the invasion of the mucosal barrier was also noted in the intranasal model. At the 5th day post-intranasal-inoculation, the bacterial counts of ΔClbP in lungs, brain, spleen, and blood of the mice were significantly less than that of the 1084S group (Fig. S3B). Besides, ΔClbP was hard to be detected in the blood, even via the intravenous inoculation route (Fig. S3C). The CFU of ΔClbP in the brain was significantly decreased at 24 hours post-intravenous-inoculation. Taken together, the deletion of *clbP* significantly attenuated *K. pneumoniae* 1084S virulence to get access to the brain in the three different meningitis mouse models.

References

Asano, K., Takahashi, N., Ushiki, M., Monya, M., Aihara, F., Kuboki, E., Moriyama, S., Iida, M., Kitamura, H., Qiu, C.H., Watanabe, T., and Tanaka, M. (2015). Intestinal CD169(+) macrophages initiate mucosal inflammation by secreting CCL8 that recruits inflammatory monocytes. *Nat Commun* 6**,** 7802.

Cougnoux, A., Delmas, J., Gibold, L., Fais, T., Romagnoli, C., Robin, F., Cuevas-Ramos, G., Oswald, E., Darfeuille-Michaud, A., Prati, F., Dalmasso, G., and Bonnet, R. (2016). Small-molecule inhibitors prevent the genotoxic and protumoural effects induced by colibactin-producing bacteria. *Gut* 65**,**

278-285.

Cougnoux, A., Gibold, L., Robin, F., Dubois, D., Pradel, N., Darfeuille-Michaud, A., Dalmasso, G., Delmas, J., and Bonnet, R. (2012). Analysis of structure-function relationships in the colibactin-maturating enzyme ClbP. *J Mol Biol* 424**,** 203-214.

Dubois, D., Baron, O., Cougnoux, A., Delmas, J., Pradel, N., Boury, M., Bouchon, B., Bringer, M.A., Nougayrede, J.P., Oswald, E., and Bonnet, R. (2011). ClbP is a prototype of a peptidase subgroup involved in biosynthesis of nonribosomal peptides. *J Biol Chem* 286**,** 35562-35570.

Fung, C.P., Lin, Y.T., Lin, J.C., Chen, T.L., Yeh, K.M., Chang, F.Y., Chuang, H.C., Wu, H.S., Tseng, C.P., and Siu, L.K. (2012). Klebsiella pneumoniae in gastrointestinal tract and pyogenic liver abscess. *Emerg Infect Dis* 18**,** 1322-1325.

Heiseke, A.F., Faul, A.C., Lehr, H.A., Forster, I., Schmid, R.M., Krug, A.B., and Reindl, W. (2012). CCL17 promotes intestinal inflammation in mice and counteracts regulatory T cell-mediated protection from colitis. *Gastroenterology* 142**,** 335-345.

Mccarthy, A.J., Martin, P., Cloup, E., Stabler, R.A., Oswald, E., and Taylor, P.W. (2015). The Genotoxin Colibactin Is a Determinant of Virulence in Escherichia coli K1 Experimental Neonatal Systemic Infection. *Infect Immun* 83**,** 3704-3711.

Mikami, Y., Dobschutz, E.V., Sommer, O., Wellner, U., Unno, M., Hopt, U., and Keck, T. (2009). Matrix metalloproteinase-9 derived from polymorphonuclear neutrophils increases gut barrier dysfunction and bacterial translocation in rat severe acute pancreatitis. *Surgery* 145**,** 147-156.

Skorupski, K., and Taylor, R.K. (1996). Positive selection vectors for allelic exchange. *Gene* 169**,** 47-52.

Trautman, E., Healy, A.R., Shine, E., Herzon, S.B., and Crawford, J.M. (2017). Domain-targeted metabolomics delineates the heterocycle assembly steps of colibactin biosynthesis. *J Am Chem Soc*.

Tu, Y.C., Lu, M.C., Chiang, M.K., Huang, S.P., Peng, H.L., Chang, H.Y., Jan, M.S., and Lai, Y.C. (2009). Genetic requirements for Klebsiella pneumoniae-induced liver abscess in an oral infection model. *Infect Immun* 77**,** 2657-2671.

Supplementary figure legends

Figure S1. Histological examination of intestinal mucosa in response to orogastric inoculation of *K. pneumoniae*. Eight-wk old BALB/c mice which were orogastrically inoculated with PBS as a control, with 1×10^8 CFU of *K. pneumoniae* 1084S or ΔClbA, were sacrificed at the 3rd day post-inoculation. Swiss rolls of the whole small intestines harvested from the control and infected mice were paraffin embedded. Representative images of hematoxylin and eosin (H/E) stained sections are presented. Scale bar in (A-C) is 2 mm; in (D-I) is 200 μm.

Figure S2. Host response induced upon the *in vivo* interaction between colibactin-producing *K. pneumoniae* and intestinal mucosa. (A) At the 3rd day post-orogastric inoculation with 1×10^8 CFU of *K. pneumoniae* 1084S or ΔClbA, mice were sacrificed and intestinal mucosa were harvested for comparative RNA-Seq analyses. Compared to ΔClbA, a total of 526 LncRNAs displayed differential expression in 1084S-infected mucosa, including 384 upregulated LncRNAs and 142 downregulated LncRNAs. **(B)** GO (Gene ontology) analysis and **(C)** KEGG (Kyoto Encyclopedia of Genes and Genomes database) pathway analysis of differently expressed LncRNAs. **(D)** Fold of activation (Log_2) of genes with predicted function in chemokines signaling pathway and leukocyte transendothelial migration.

Figure S3. Deletion of *clbP* significantly attenuated *K. pneumoniae* 1084S virulence in mouse meningitis models. The *clbP* deletion mutant, ΔClbP, was inoculated into five to six 8-wk-old BALB/c male mice through the orogastric, intranasal, and intravenous route at inoculums of 1×10^8, 1×10^6, and 1×10^6 CFU, respectively. All mice of the ΔClbP-inoculated survived the course of the experiment. **(A)** At the 7th day post-orogastric-inoculation, stool, intestinal mucosa, liver, spleen, brain, and blood of the ΔClbP-inoculated mice were retrieved for enumeration of CFU. **(B)** Bacterial loads in lungs, brain, spleen, and blood which were harvested from the mice intranasally inoculated with 1×10^6 CFU of ΔClbP. **(C)** Bacterial loads in brain and blood retrieved at 24 hours post-intravenous-inoculation with 1×10^6 CFU of ΔClbP. CFU per gram of tissues of the ΔClbP group (slash) is plotted in comparison to that of the 1084S (dark gray) and ΔClbA (white) group and presented in box and whisker. The edges of each box are the 25th and 75th percentiles, and the middle is the median. An asterisk (∗) represents statistical significance of $CFUg^{-1}$, $p<0.05$ (one-tailed) determined by Mann-Whitney test, between 1084S and ΔClbP.

Fig. S1

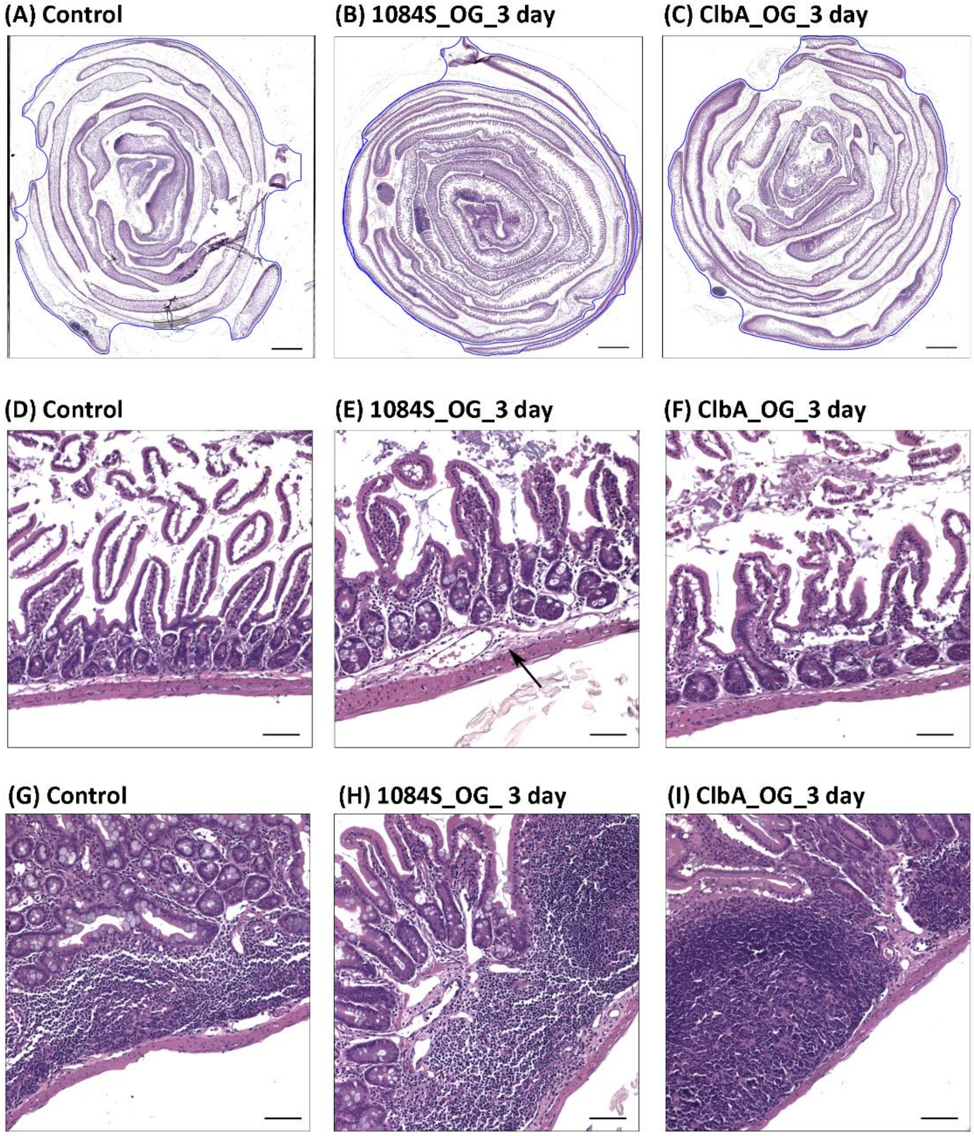

(A) Control
(B) 1084S_OG_3 day
(C) ClbA_OG_3 day
(D) Control
(E) 1084S_OG_3 day
(F) ClbA_OG_3 day
(G) Control
(H) 1084S_OG_ 3 day
(I) ClbA_OG_3 day

Fig. S2

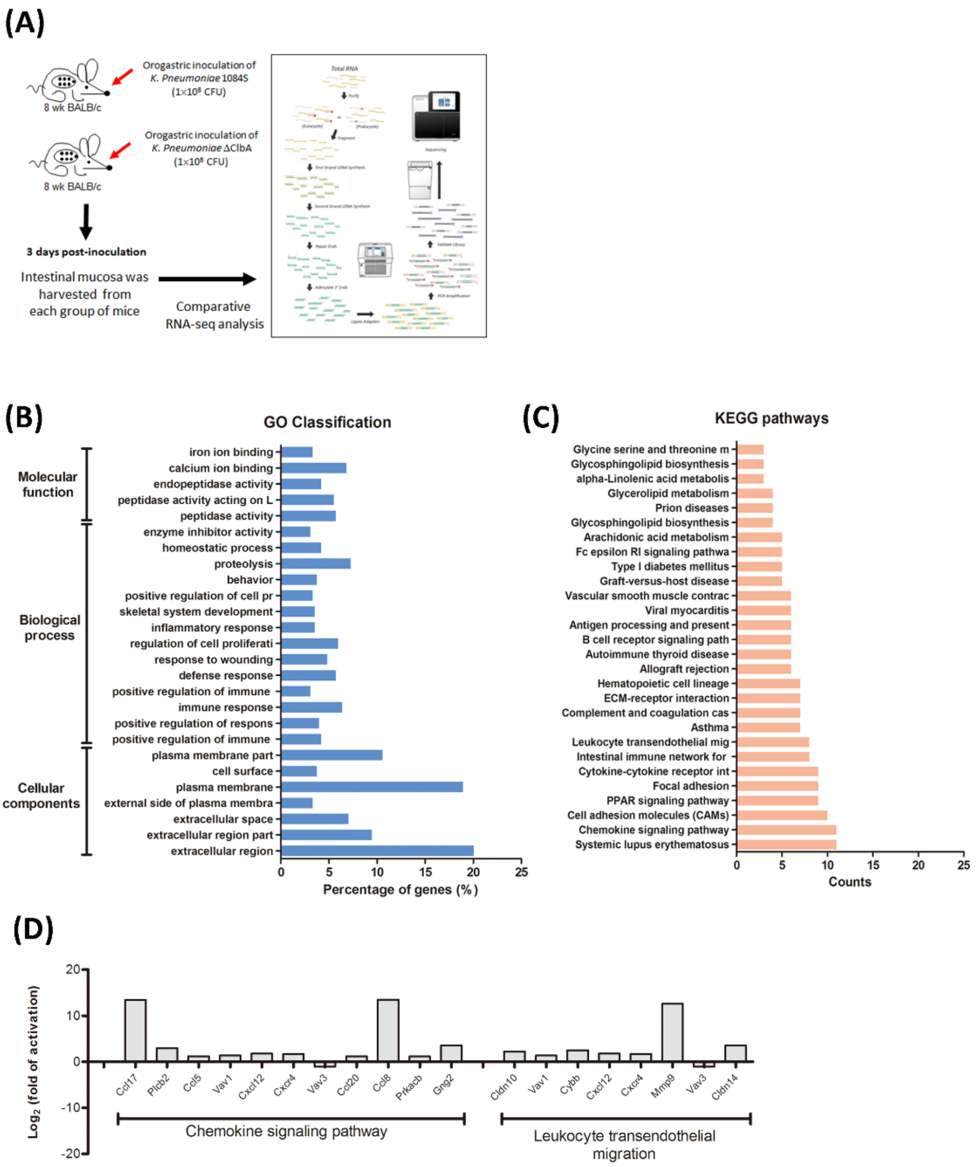
(A)
Orogastric inoculation of K. Pneumoniae 1084S (1×10⁸ CFU)
8 wk BALB/c
Orogastric inoculation of K. Pneumoniae ΔClbA (1×10⁸ CFU)
8 wk BALB/c
3 days post-inoculation
Intestinal mucosa was harvested from each group of mice
Comparative RNA-seq analysis
(B)
GO Classification
Molecular function
Biological process
Cellular components
iron ion binding
calcium ion binding
endopeptidase activity
peptidase activity acting on L
peptidase activity
enzyme inhibitor activity
homeostatic process
proteolysis
behavior
positive regulation of cell pr
skeletal system development
inflammatory response
regulation of cell proliferati
response to wounding
defense response
positive regulation of immune
immune response
positive regulation of respons
positive regulation of immune
plasma membrane part
cell surface
plasma membrane
external side of plasma membra
extracellular space
extracellular region part
extracellular region
0
5
10
15
20
25
Percentage of genes (%)
(C)
KEGG pathways
Glycine serine and threonine m
Glycosphingolipid biosynthesis
alpha-Linolenic acid metabolis
Glycerolipid metabolism
Prion diseases
Glycosphingolipid biosynthesis
Arachidonic acid metabolism
Fc epsilon RI signaling pathwa
Type I diabetes mellitus
Graft-versus-host disease
Vascular smooth muscle contrac
Viral myocarditis
Antigen processing and present
B cell receptor signaling path
Autoimmune thyroid disease
Allograft rejection
Hematopoietic cell lineage
ECM-receptor interaction
Complement and coagulation cas
Asthma
Leukocyte transendothelial mig
Intestinal immune network for
Cytokine-cytokine receptor int
Focal adhesion
PPAR signaling pathway
Cell adhesion molecules (CAMs)
Chemokine signaling pathway
Systemic lupus erythematosus
0
5
10
15
20
25
Counts
(D)
Log₂ (fold of activation)
20
10
0
-10
-20
Ccl17
Plcb2
Ccl5
Vav1
Cxcl12
Cxcr4
Vav3
Ccl20
Ccl8
Prkacb
Gng2
Cldn10
Vav1
Cybb
Cxcl12
Cxcr4
Mmp9
Vav3
Cldn14
Chemokine signaling pathway
Leukocyte transendothelial migration

Fig. S3

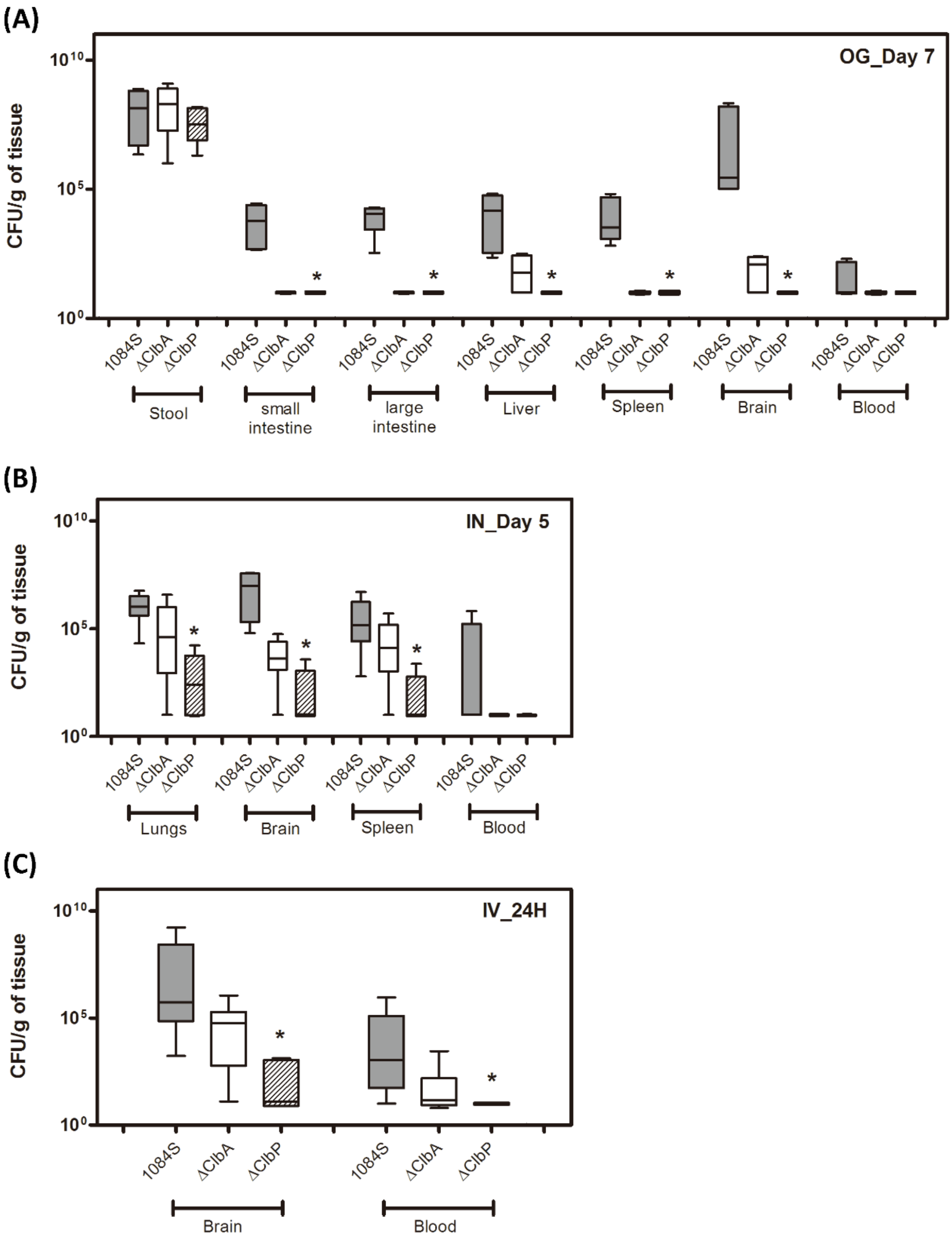
(A)
OG_Day 7
CFU/g of tissue
10¹⁰
10⁵
10⁰
1084S
ΔClbA
ΔClbP
Stool
small intestine
large intestine
Liver
Spleen
Brain
Blood
(B)
IN_Day 5
CFU/g of tissue
Lungs
Brain
Spleen
Blood
(C)
IV_24H
CFU/g of tissue
Brain
Blood
