# Supplementary figures and images for: Colibactin Contributes to the Hypervirulence of pks+ K1 CC23 Klebsiella pneumoniae in Mouse Meningitis Infections
Source: Front Cell Infect Microbiol. 2017 Mar 31;7:103. doi: 10.3389/fcimb.2017.00103 (PMC5374149; doi:10.3389/fcimb.2017.00103)

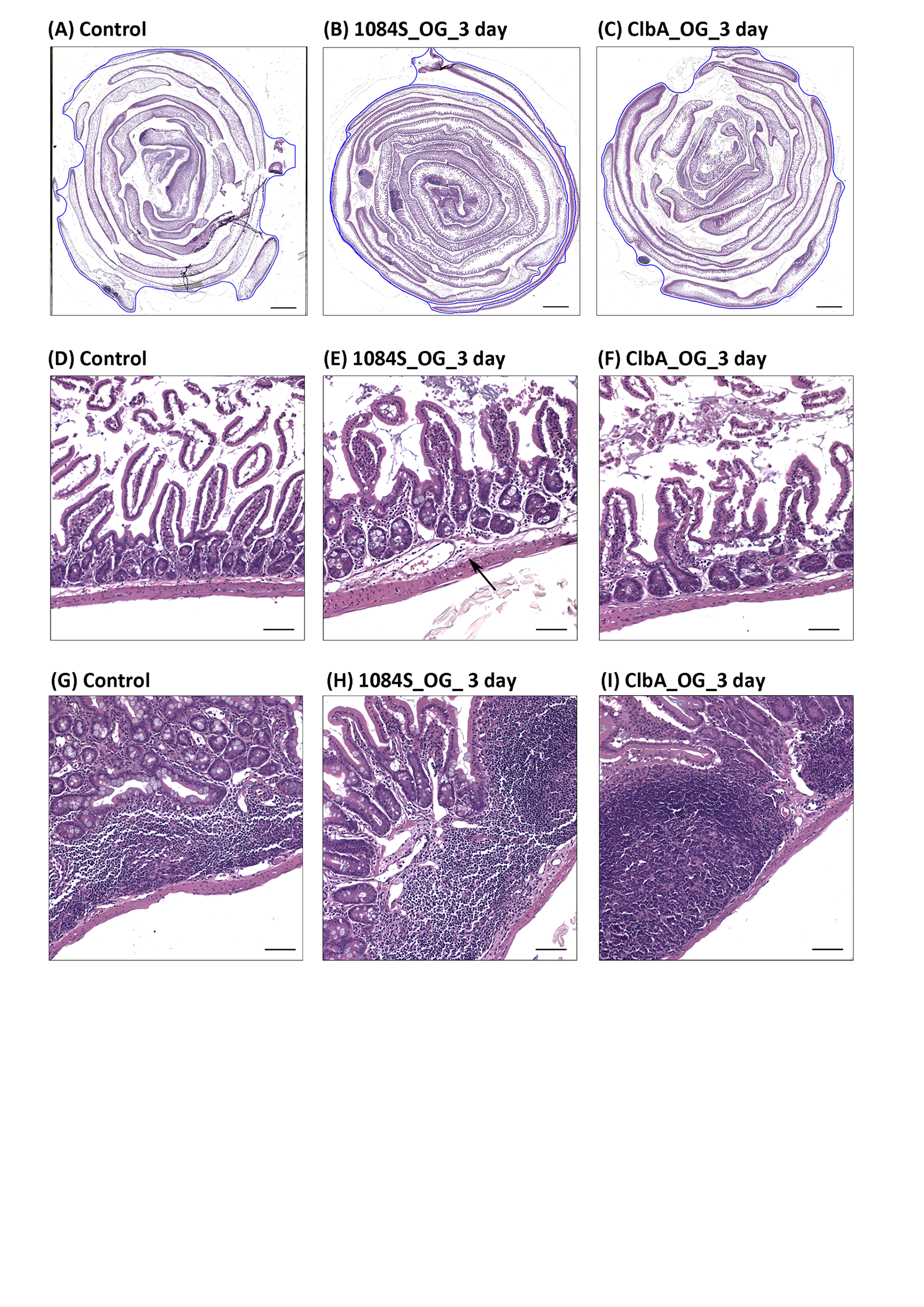

Supplement: Supplementary file 4 [file Image1.TIF]

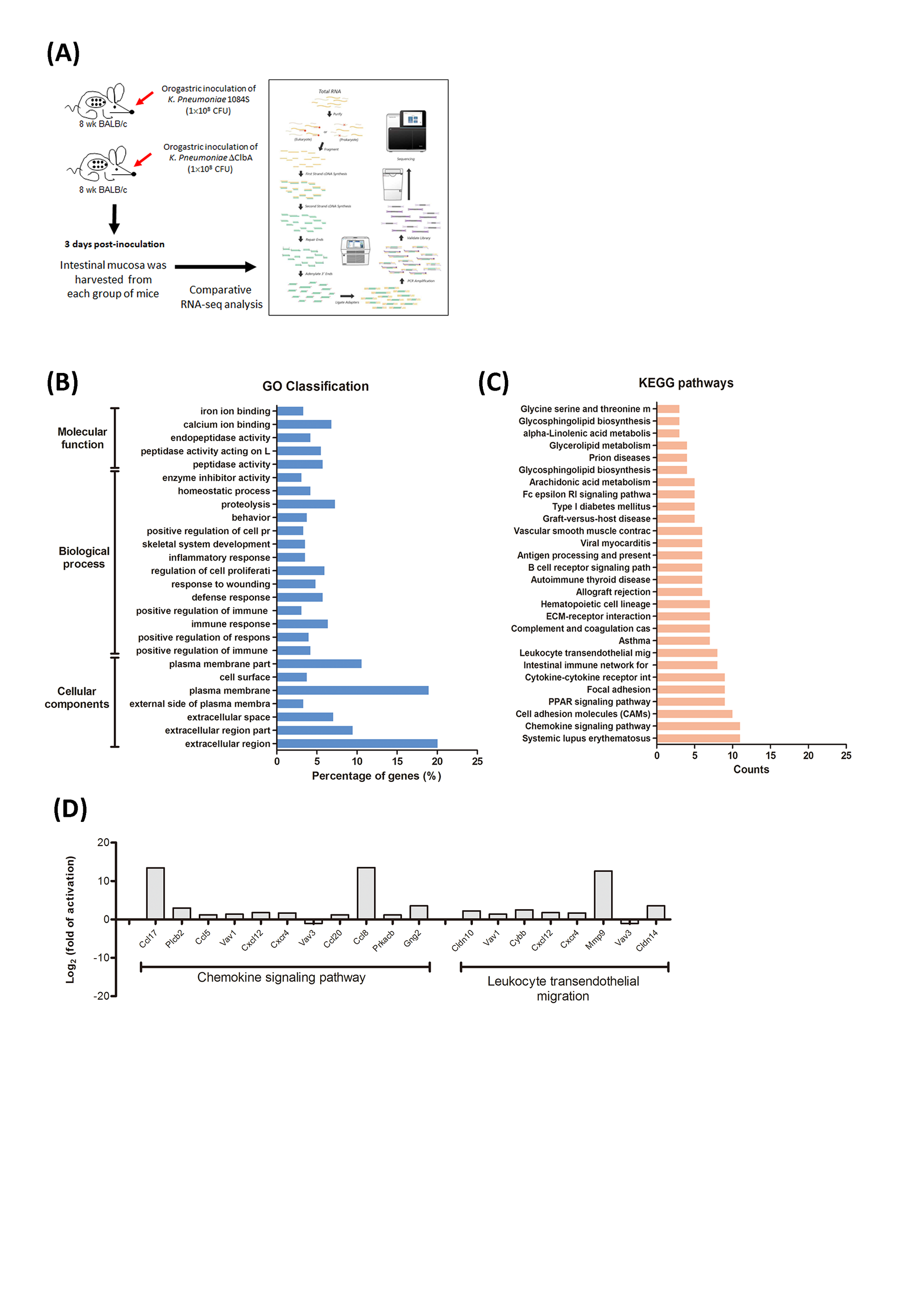

Supplement: Supplementary file 5 [file Image2.TIF]

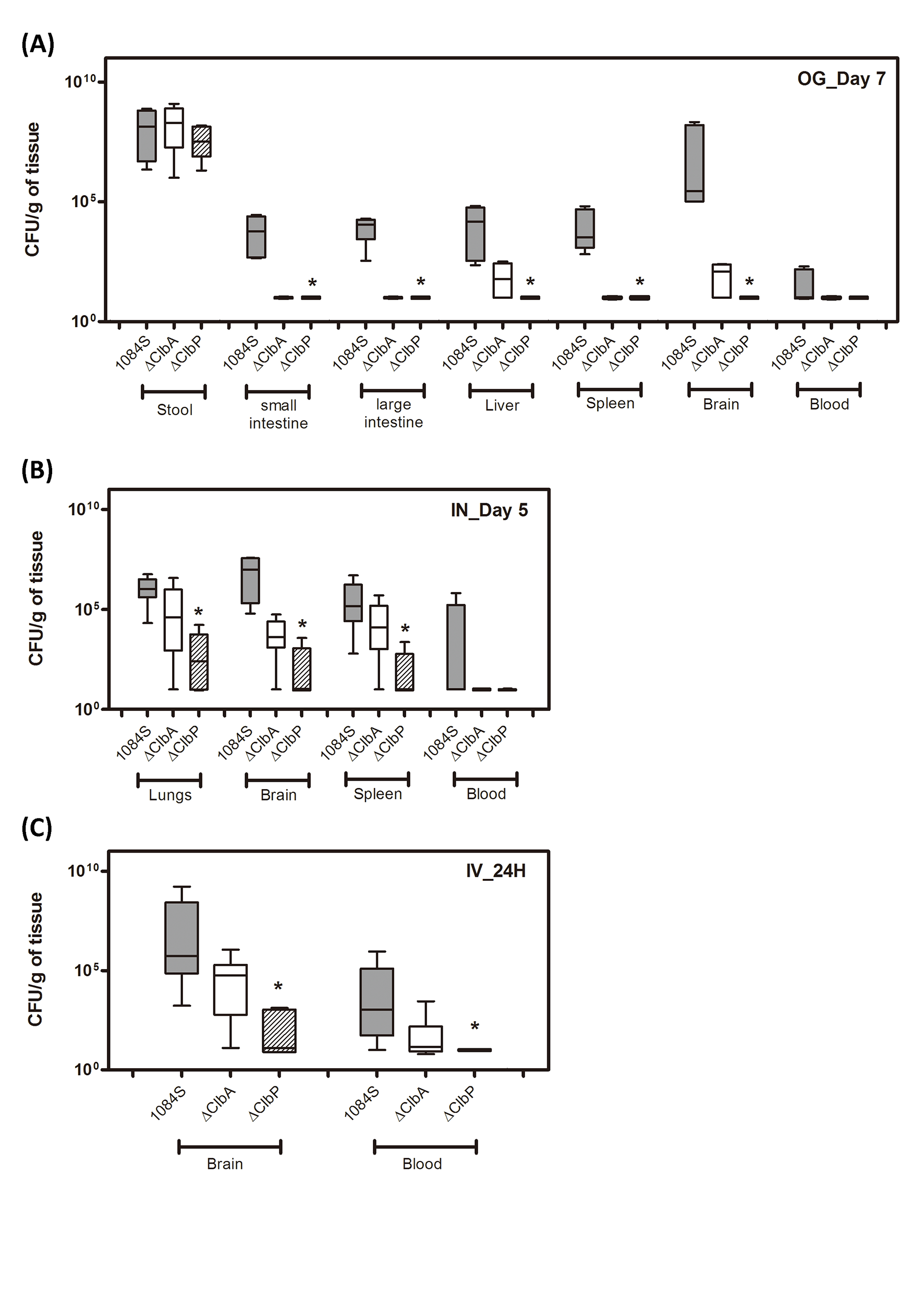

Supplement: Supplementary file 6 [file Image3.TIF]
